# Copy number alterations in pediatric B-cell precursor acute lymphoblastic leukemia patients and their association with patients’ outcome

**DOI:** 10.1007/s00277-024-06102-2

**Published:** 2024-11-26

**Authors:** Nesma E. Abdelfattah, Ghada M. Elsayed, Amira H. Soliman, Emad N. Ebeid, Mona S. El Ashry

**Affiliations:** 1 Clinical Pathology Department, National Cancer Institute, Cairo University, Kasr Al Eini Street, 4 Form El Khalig, Cairo, 11796 Egypt; 2 Pediatric Oncology Department, National Cancer Institute, Cairo University, Cairo, Egypt

**Keywords:** ALL, BCP-ALL, *PAX5*, *RUNX1*, MLPA, FISH

## Abstract

Genetic abnormalities provide diagnostic and prognostic information for pediatric B-cell precursor acute lymphoblastic leukemia (BCP-ALL) patients. The aim of this study was to determine the effects of genetic CNAs and *RUNX1* gene abnormalities on the outcome of pediatric BCP-ALL patients. This study included 78 de novo-BCP-ALL pediatric patients who presented to the Pediatric Oncology Department of the National Cancer Institute (NCI), Cairo University. We aimed to study the impact of copy number alteration (CNA) of 8 of the most altered genes in BCP-ALL patients, in addition to *RUNX1* gene abnormalities, on patient survival and response to treatment. Multiplex ligation-dependent probe amplification (MLPA) was used to detect CNA, while *RUNX1* gene alterations were detected by fluorescence in situ hybridization (FISH). CNA of the *PAX5* gene was significantly associated with worse overall survival (OS) and event-free survival (EFS) (*P = 0.012 and P = 0.025*, respectively). An increase in the CNA of *ETV6* was associated with an increase in minimal residual disease (MRD) on day 15 (P = 0.041). Although *RUNX1* gene abnormalities were not a predictor of shorter OS or EFS, an interesting significant association was found between *PAX5* CNA and *RUNX1* gene gain and translocation (P = 0.017 and P = 0.041, respectively). *PAX5* CNA is an adverse prognostic factor. *ETV6* CNA is associated with high MRD on day 15.

**Supplementary Information:**

The online version contains supplementary material available at 10.1007/s00277-024-06102-2.

## Introduction

Acute lymphoblastic leukemia (ALL) is the most common type of blood cancer in the pediatric population, representing 75–80% of acute leukemias among children, with 80–85% of acute leukemias being of the B-cell phenotype [1].

Genetic abnormalities in BCPC-ALL patients affect patient outcomes and therefore are used in risk stratification for treatment [2]. Approximately 75% of BCP-ALL patients with recurrent genetic translocations are between 1 and 9 years of age, 35% are between 10 and 20 years of age, and 40% are older than 20 years [3]. The outcomes of BCP-ALL patients differ by genetic subtype, i.e., ETV6::RUNX1(E/R), high hyperdiploidy, and TCF3::PBX1 patients have favorable prognoses, while BCR::ABL1*-* and *KMT2A*-rearranged BCP-ALL patients are associated with unfavorable treatment outcomes. Approximately 25% of patients have a genetically unclassified disease, which is defined as ‘B-other’ [4].

ETV6::RUNX1 (E/R) is the most common fusion gene in childhood ALL. Although it was understood from the data available at that time that this type of BCP-ALL has a favorable outcome based on the response to treatment and clinical response, 20% of patients experience late relapse [5]. Relapses occur in most patients several years after therapy ends and sometimes after 10–20 years, while early recurrence accounts for only 10% of E/R-positive relapses [6]. The genome of E/R-positive ALL has been thoroughly characterized at both the CNA and cytogenetic levels. Molecular or traditional cytogenetic techniques have been used to detect a considerable number of secondary genetic abnormalities in E/R-positive ALL patients [7, 8]. Several driver CNAs are necessary for the development of the leukemia E/R fusion gene, which is the first step in leukemia transformation [9]. The pathogenesis of E/R positive BCP-ALL is believed to imply a “two-hit” model, whereby E/R rearrangement is followed by a series of secondary mutations that trigger overt leukemia [9]. Although there may not be an exclusive second genetic “hit,” most patients with E/R-positive ALL have deletions of the normal *ETV6* allele at the time of diagnosis. Other genes encoding transcription factors that are important for B-cell differentiation and maturation, such as *PAX5*, *EBF1* and *IKZF1*, have also been found to frequently have deletions in cases of E/R-positive ALL [10].

On the other hand, the *RUNX1* gene participates in a distinct cytogenetic subgroup of BCP-ALL, defined as intrachromosomal amplification of chromosome 21 (iAMP21), which was originally described as multiple copies of the *RUNX1* gene on a structurally abnormal chromosome 21. This category includes 2% of childhood ALL patients and has a relatively poor prognosis [11].

Other genetic alterations may occur in BCP-ALL either as CNAs or specific mutations. CNAs occur within a considerable proportion of pediatric patients with BCP‑ALL, and they have been proven to have prognostic significance; for example, *PAX5* alterations are observed in most subtypes of BCP-ALL and are likely fundamental to the pathogenesis of the disease [1].

*PAX5* alt BCP-ALL is characterized by diverse *PAX5* alterations, including rearrangements (most commonly with *ETV6* or *NOL4L*), sequence mutations, and intragenic amplification. *PAX5* p.Pro80Arg is characterized by a universal p.Pro80Arg mutation with deletion or mutation of the remaining allele and alterations in the Ras and *JAK-STAT* pathway genes. Patients with *PAX5*alt or *PAX5* p.Pro80Arg BCP-ALL have an intermediate prognosis [12].

MLPA is a sensitive method based upon multiplex polymerase chain reaction (PCR) and capillary electrophoresis. It has the advantages of low price and short turn-around time in the detection of important copy number changes in the most frequently altered gene loci in BCP-ALL, namely, *EBF1*,* IKZF1*,* PAX5*,* CDKN2A/B*,* ETV6*,* BTG1*,* RB1*, and *PAR1* (for the detection of *P2RY8-CRLF*), in the *Xp22.33/Yp11.32 region*. These genes participate in B-cell differentiation, cell cycle regulation, transcription, proliferation, and cell survival in BCP-ALL [13, 14].

The aim of this study was to determine the effects of genetic CNAs and *RUNX1* gene abnormalities on the outcome of pediatric BCP-ALL patients.

## Patients and methods

The present study included seventy-eight newly diagnosed pediatric BCP-ALL patients who were diagnosed between December 2018 and December 2020. All patients were referred to the outpatient clinic of the Pediatric Oncology Department of the National Cancer Institute (NCI), Cairo University.

We used the French American-British (FAB) and WHO Classification of Tumors of Hematopoietic and Lymphoid Tissues criteria of 2016 for the diagnosis of Pediatric BCP-ALL [15]. Diagnostic bone marrow (BM) samples were collected at presentation before receiving any treatment. The diagnosis of Pediatric BCP-ALL was based on peripheral blood (PB) and BM morphology, cytochemistry, immunophenotyping (IPT), conventional cytogenetic analysis, fluorescence in situ hybridization (FISH) and reverse transcription polymerase chain reaction (RT‒PCR).

### Flow cytometry analysis

IPT was performed on BM and/or PB samples to confirm the diagnosis of ALL using a fluorescein-labeled mouse monoclonal antibody panel of lymphoid markers (CD10 and CD19 for the B lymphoid series; CD3, CD2, CD4, CD8, CD7 and CD5 for the T lymphoid series) and the stem cell marker CD34, in addition to HLA-DR, on a routine basis.

### Cytogenetic analysis

Conventional karyotyping was performed on a minimum of 20 G-banded metaphases on patients’ initial BM samples. We used an IKAROS imaging system (Metasystems, Altlussheim, Germany) for the analysis of metaphases. *RUNX1* gene analysis was performed using XL *RUNX1* Break Apart Probe (Ref. No. D-5096-100-OG) to study *RUNX1* gene alterations in the form of amplifications, deletions or translocations according to the manufacturer’s instructions. A minimum of 10 metaphases and 200 interphase nuclei were analyzed using a fluorescence microscope (AxioImager. Z1 mot, Carl Zeiss Ltd., Hertfordshir, UK). Image capture and processing were performed using an ISIS imaging system (Metasystems, Altlussheim, Germany).

### Recurrent translocations

Reverse transcription PCR was performed for all samples for the detection of the fusion transcripts t(9;22) (q34;q11), t(4;11)(q21;q23), t(12;21)(p13;q22) and t(1;19)(q23;p13).

### MLPA

MLPA was performed on the samples of 70 patients due to limited resources. DNA extraction was performed using a QIAGEN QIAamp DNA Blood Mini Kit (100), followed by the detection of genetic CNAs using a SALSA MLPA P335 Kit (MRC-Holland, Amsterdam, the Netherlands) according to the manufacturer’s instructions. The PCR fragments were separated by capillary electrophoresis on a Life Technologies 3,500 Genetic Analyzer (Thermo Fisher Scientific, Waltham, MA, USA). MLPA data were analyzed using Coffalyser. Net v.140721.1958 (MRC-Holland, Amsterdam, Netherlands). Normal copy number was defined by a final ratio (FR) of 0.80 < FR < 1.20, homozygous deletion by FR = 0, heterozygous deletion by 0.40 < FR < 0.65, and heterozygous duplication by 1.30 < FR < 1.65, and ambiguous copy number was defined by all other values.

### Treatment protocol

All children were treated according to the treatment protocol followed at NCI, Cairo University, which is adopted from the Saint Jude Total XV protocol (TXV). The treatment protocol consisted of three phases.

1-Induction of remission (first 42 days): Patients who received 6 chemotherapeutic agents (vincristine, adriamycin, L-asparaginase, cyclophsphamide, cytarabine and 6-mercaptopurine) in addition to steroids, with BM evaluation at days 15 and 42 (the end of induction). These evaluations are milestones that are common in determining patient risk stratification, pathways, and further management.

2-Consolidation: Four courses of high-dose methotrexate and 6-mercaptopurine.

3-Maintenance: 120 weeks of chemotherapy with evaluation at week 7 and week 48. Complete remission (CR) was defined as no circulating lymphoblasts or extramedullary disease, trilineage hematopoiesis, less than 5% blasts in the BM, absolute neutrophil counts (ANCs) greater than 1.0 × 10^9^/L, and platelet counts greater than 100 × 10^9^/L. MRD was defined as the presence of leukemic cells above the threshold of detection by multicolor flow cytometry on day 15 and day 42, at a cutoff of 1 and 0.01, respectively [16].

OS was calculated from the date of diagnosis until the date of death or last follow-up. EFS was calculated from the date of complete remission until the date of relapse, death, or last follow-up.

### Statistical analysis

Data management and analysis were performed using the Statistical Package for Social Sciences (SPSS) version 23. Numerical data were summarized using means and standard deviations or medians and ranges, as appropriate. Categorical data are summarized as numbers and percentages.

The chi-square test or Fisher’s test was used to compare categorical data between independent groups. The normality of the numeric variables was evaluated using Shapiro‒Wilk and Kolmogrov‒Smirnov tests, and all variables were found to be not normally distributed and compared between two or three groups using the Mann‒Whitney U test or Krusksl‒Wallis test, respectively. Post hoc tests were performed for non-parametric Krusksl Wallis test was done using the Dunn’s test. Post hoc for chi square test was done using Bonferroni adjustment. Post hoc test for survival was done using log rank (mantel-cox) test.

OS and EFS were estimated using the Kaplan‒Meier method. Survival curves were compared with the log-rank test. Multivariate analysis was performed with a Cox regression model. Hazard ratios (HRs) and 95% confidence intervals (CIs) were calculated. A significant CI does not crossover 1.

Multivariate logistic regression analysis was performed to predict MRD15 and MRD42. Odds ratios (ORs) and 95% CIs were calculated. A *P* value less than or equal to 0.05 was considered significant. All tests were two tailed.

## Results

### Initial patient characteristics

Among the seventy-eight pediatric BCP-ALL patients, the median age was 6 years; 47 (60.3%) were males, while 31 (39.7%) were females. Fifty-five (70.5%) patients were presented with hepatomegaly, 59 (75.6%) with splenomegaly, and 51 (65.4%) with lymphadenopathy, while only 6 (7.7%) patients were positive for central nervous system (CNS) involvement. Fifty-four patients (69.2%) had fever, 68 (87.2%) had anemia, and thirty patients (38.5%) experienced bleeding at presentation.

Using IPT, 4 (5.1%) patients were subclassified as having pro-B ALL, 33 (42.3%) had common ALL, and 41 (52.6%) had the pre-B phenotype Sixty-eight patients were subjected to cytogenetics analysis (karyotyping). The median (range) modal chromosome number (MCN) was 46 (33 to 61). We found Normal karyotype in 13 (19%) patients, and abnormal karyotype in 55 (81%) patients. We detected Numerical abnormalities in 43 (63.2%) patients. We applied Genetic risk stratification for 70/78 (89.7%) ((Whenever the numerical abnormalities could not be detected by conventional cytogenetics the DNA index was used instead), the details of the cytogenetic analysis results and cytogenetic risk stratification are listed in Table 1.

### Treatment protocol and response to induction

Seventy-four patients (94.9%) received the TXV protocol (TVX-HDMTX), while 4 (5.1%) followed the infantile protocol ± administered steroid therapy. Three (3.8%) patients received tyrosine kinase inhibitors, while one (1.3%) patient underwent Hematopoietic stem cell transplantation (Allogenic) using a graft from matched sibling. We followed Seventy-two 72/78 patients (92.3%) during induction and 6/78 (7.6%) patients died during induction. Using flow cytometry, the patients’ response to induction therapy was assessed at days 15 and day 42 for 69/72 (95.8%) and 57/72 (79.1) %, respectively. Some data were missing from medical records on day 15 and day 42, Table 1.

70 of 72 (97.2%) achieved CR, while 2 (2.8%) patients were refractory; the median MRD on day 15 was 0.50 (0 to 88), and the median MRD on day 42 was 0.01 (0.01 to 40). We applied NCI risk stratification for 77/78 (98.7%) patients, we were not certain of the clinical data for one patient who we could not apply the NCI risk stratification (initial CNS involvement), Table 1. NCI risk classification was done using the age, initial TLC, molecular and cytogenetic results and response to induction, 37 (48.1%) patients were in the standard risk (SR) group, 26 (33.8%) patients in the low risk (LR) group and 11 (14.3%) patients in the high-risk group, while 3 (3.9%) patients younger than one year of age were excluded from this risk stratification. Baseline demographic data, laboratory characteristics, risk stratification and response to treatment are shown in Table 1.

**Table 1:** Initial patient’s characteristics

Data	*n* = 78 n (%)	Data	*n* = 78 n (%)
Age (Median and Range), years	6 (0.08-17)*	t(4;11) (q21;q23)	1 (1.3)
Sex		t(1;19) (q23;p13.3)	4 (5.1)
Males	47 (60.3)	t(9;22) (q34.1;q11.2)	4 (5.1)
Females	31 (39.7)	t(12;21) (p13.2;q22.1)	11 (14.1)
Peripheral blood		*iAMP 21* (*n* = 78)	2 (2.6)
TLC x 10^9^/L	36 (1, -719) *	**NCI risk stratification**	(*n* = 77)
Platelets x 10^9^/L	35.5 (6-276) *	SR	37 (48.1)
Hemoglobin (g/dl) Mean ± SD	7.45 ± 2.56	LR	26 (33.8)
Peripheral Blood blasts (%)	80 (12–98) *	HR	11 (14.3)
Bone Marrow blasts (%)	93 (25–100) *	Infantile	3 (3.9)
IPT		**Genetic risk stratification**	(*n* = 70)
Pre-B	41(52.6)	Favorable	32 (45.7)
Pro-B	4 (5.1)	Intermediate	27 (38.6)
CALL	33 (42.3)	Adverse	11 (15.7)
CD34 positive	40 (51.3)	**MRD D15 (n = 69)**	0.50 (0–88) *
Aberrant myeloid markers	6 (7.7)	MRD D15 > 1	20 (28.9%)
DNA index	1 (1-1.34) *	**MRD D42 (n = 57)**	0.01 (0.01-40)*
Cytogenetics (*n* = 68)		MRD D42 > 0.01	15 (26.3%)
MCN	46 (33–61) *	**Complete remission**	**(n = 72)**
Hypodiploid	15 (22.1)	No	2 (2.8)
Diploid	25 (36.8)	Yes	70 (97.2)
Hyperdiploid	7 (10.2)	**Relapse**	4 (5%)
High hyperploid	21 (26.9)	**Death**	23(29.5%)
Complex	15 (22.1)		
Recurrent translocations			

*Note* Data are presented as number (percentage) unless otherwise indicated. * Shown as the median value with the range in between brackets. Abbreviations: NCI: National Cancer Institute, IPT: Immuno-phenotyping, TLC: Total leucocytic count

### RUNX1 gene abnormalities testing by FISH

We investigated all patients (78 patients) for the detection of recurrent translocations using RT-PCR on routine basis. We detected ETV6::RUNX1*(E/R)* translocation in 11/78 (14.1%) patients. We used FISH for the investigation of RUNX1 gene abnormalities in seventy-seven patients. We have lost one heparinized bone marrow sample for a patient whose extracted DNA was available for MLPA testing. We have detected *RUNX1* gene abnormalities in 51 (66.2%) patients. The most common abnormality was *RUNX1* gain in 33 (42.9%) patients, followed by *RUNX1* translocation in 19 (24.7%), *RUNX1* deletion in 7 (9.1%) and *iAMP* in 21 − 2 (2.6%) patients (Supplementary Fig. 2). Among the 19 patients who were found to have RUNX 1 translocations by RT/PCR, 11 had the *ETV6* (12p13) gene as a partner gene, while in the remaining 8 patients, the partner gene could not be confirmed by another method. Considering the results of the two mentioned tests (RT/PCR and XL RUNX1break apart FISH probe) we have used to investigate the *RUNX1* gene abnormalities, we concluded that we have 19 patients with *RUNX1* translocations 11 of them the partner gene was confirmed to be *ETV6* using RT/PCR while in the remaining 8 patients, the partner gene partner gene for RUNX1 translocation could not be confirmed.

### Copy number alterations (CNAs)

**Fig. 1:**
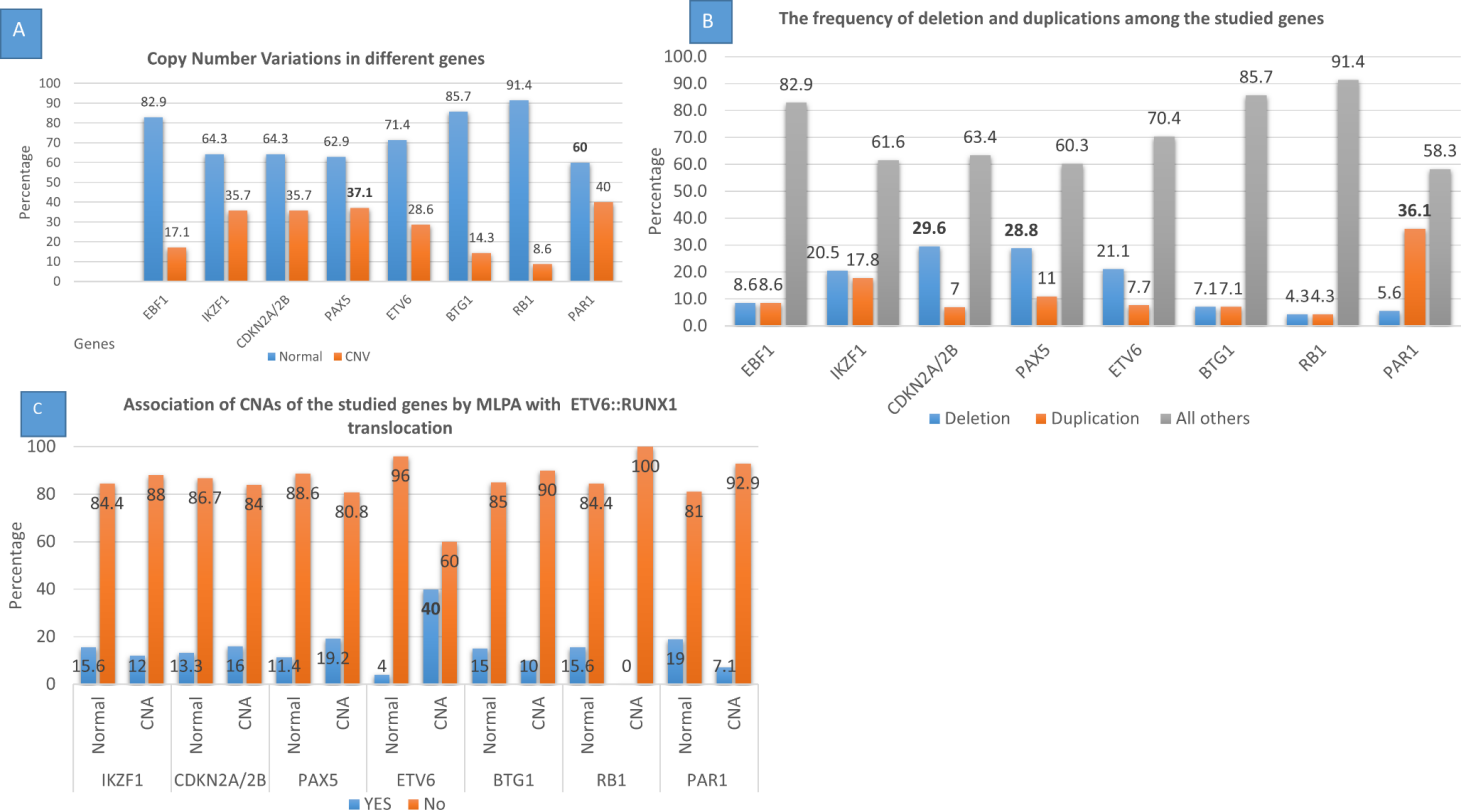
**A**: Frequency of copy number alteration of the studied genes by MLPA among the whole group of patients demonstrating that the most detected gene with CNAs was *PAX5* gene. **B**: The frequency of deletions and duplications in the studied genes showing that *CDKN2A/2B* is the most deleted gene in our study while the most duplicated one was *PAR1* complex (*Xp22.33 / Yp11.32* region) **C**: Association of copy number alterations in the studied genes by MLPA with the ETV6::RUNX1 translocation showing that this translocation is significantly associated with CNAs in *ETV6* gene. * All other: cases with normal copy number and cases with ambiguous copy number

We studied the CNAs of the *EBF1*,* IKZF1*,* PAX5*,* CDKN2A/B*,* ETV6*,* BTG1*,* RB1*, and PAR1 complex genes. Sixty out of 70 (85.7%) patients had at least one CNA in at least one of the studied genes. Twenty-two (31.4%) patients had deletions (heterozygous and homozygous deletions), 18 (25.7%) patients had heterozygous duplications, and 20 (28.6%) patients had coexistence of deletion and duplication in different genes. The most frequently deleted genes were *CDKN2A*/2B and *PAX5* in 21 (29.2%) and 21 (28.8%) patients, respectively. In contrast, the most frequently duplicated gene was the *Xp22.33/Yp11.32* region in 26 (36.1%) children (Table 2, Fig. 1and Supplementary Fig. 1). Supplementary Table (SI 1–4) Show the frequency of CNAs in different exons of the studied genes. A significant association was found between CNA in the *PAX5* gene and CNA in either the *IKZF1* gene or the *CDKN2A/2B* gene (CI = 1.622–13.249, 1.250–9.800, respectively) (Table 3). We studied the genetic CNAs in 11 E/R-positive patients, and we have found that 9 (81%) patients had CNAs in the ETV6 gene and this group of patients (*P* < 0.001) (Fig. 1 and Supplementary Fig. 3).

**Table 2:** Frequency of significant deletions and duplications in different studied genes (without addition of mixed case) among 70 BCP-ALL pediatric patients

Gene	EBF1 5q33.3	IKZF1 7p12.2	CDKN2A/ CDKN2B, (9p21.3)	PAX5 (9p13.2)	ETV6 (12p13.2 )	BTG1 (12q21.33)	RB1 (13q14.2)	Xp22.33 / Yp11.32 region
Deletion	6 (8.6)	15 (20.5)	21 (29.6)	21 (28.8)	15 (21.1)	5 (7.1)	3 (4.3)	4 (5.6)
Heterozygous deletion	6 (7.7)	15 (20.5)	20 (28.2)	21 (28.8)	14 (19.7)	5 (7.1)	3 (4.3)	1 (1.4)
Homozygous deletion	0 (0.0)	0 (0.0)	1 (1.4)	0 (0.0)	1 (1.4)	0 (0.0)	0 (0.0)	3 (4.2)
Heterozygous duplication	6 (7.7)	13 (17.8)	5 (7)	8 (11.0)	6 (8.5)	5 (5.1)	3 (4.3)	26 (36.1)
All others*	58 (82.9)	45 (61.6)	45 (63.4)	44 (60.3)	50 (70.4)	60 (85.7)	64 (91.4)	42 (58.3)
Total** (100%)	70	73	71	73	71	70	70	72

*All other: cases with normal copy number and cases with ambiguous copy number (non-significant deletion or duplication) **Total = total number of studied cases (70) + number of mixed cases Mixed: cases with significant deletion and significant duplication in different exons of the same gene so each of them are counted twice once with the cases with significant deletion and once with cases of significant duplication.Data are presented as numbers (percentages) unless otherwise indicated

**Table 3:** Coexistence of Copy Number alterations in different genes

Coexistence of genes	*n* (%)	Odds ratio	Confidence Interval
*EBF1-IKZF1*	6 (8.6)	2.053	0.584–7.218
*EBF1-CDKN2A/AB*	4 (5.7)	0.881	0.237–3.279
*EBF1-PAX5*	6 (8.6)	1.900	0.542–6.661
*EBF1-ETV6*	5 (7.1)	2.048	0.564–7.434
*EBF1-BTG1*	4 (5.7)	4.333	0.998–18.807
*EBF1-RB1*	1 (1.4)	0.964	0.102–9.080
*EBF1- Xp22.33 / Yp11.32 region*	7 (10.0)	2.467	0.695–8.752
*IKZF1-CDKN2A/2B*	12 (17.1)	2.272	0.823–6.273
*IKZF1-PAX5*	15 (21.4)	4.636	1.622–13.249
*IKZF1-ETV6*	7 (10.0)	0.957	0.323–2.834
*IKZF1-BTG1*	5 (7.1)	2.000	0.518–7.721
*IKZF1-RB1*	4 (5.7)	4.095	0.694–24.182
*IKZF1- Xp22.33 / Yp11.32 region*	9 (12.9)	0.770	0.281–2.110
*CDKN2A/2B-PAX5*	14 (20.0)	3.500	1.250–9.800
*CDKN2A/2B-ETV6*	6 (8.6)	0.699	0.230–2.130
*CDKN2A/2B–BTG1*	5 (7.1)	2.000	0.518–7.721
*CDKN2A/2B–RB1*	2 (2.9)	0.891	0.151–5.246
*CDKN2A/2B– Xp22.33 / Yp11.32 region*	8 (11.4)	0.588	0.211–1.640
*PAX5-ETV6*	10 (14.3)	2.125	0.737–6.127
*PAX5-BTG1*	6 (8.6)	3.000	0.759–11.857
*PAX5-RB1*	2 (2.9)	0.833	0.142–4.898
*PAX5- Xp22.33 / Yp11.32 region*	14 (20.0)	2.500	0.921–6.785
*ETV6-BTG1*	5 (7.1)	3.000	0.762–11.811
*ETV6-RB1*	2 (2.9)	1.278	0.215–7.598
*ETV6- Xp22.33 / Yp11.32 region*	8 (11.4)	1.000	0.347–2.882
*BTG1-RB1*	1 (1.4)	1.222	0.128–11.710
*BTG1- Xp22.33 / Yp11.32 region*	5 (7.1)	1.609	0.419–6.171
*RB1- Xp22.33 / Yp11.32 region*	1 (1.4)	0.274	0.030–2.482

*Confidence interval (CI): is considered significant if it does not enclose the value reflecting ‘no effect’, this represents a difference that is statistically significant

### Minimal residual disease (MRD)

We studied the factors that may affect MRD on day 15 and found a significant association between CNAs of *ETV6* exon 1 and exon 1 A and high MRD on day 15. In exon 1. Most patients with a normal CN for exon 1 and exon 1 A of the *ETV6* gene had significantly lower MRD on day 15 (*P* = 0.041, 0.033), as 73.2% and 72.2%, respectively, of them had MRD at days 15 ≤ 1 (Table 4). MRD on day 42 (end of induction) was found to be significantly associated with an initial total leucocyte count (TLC) less than 30,000/µl and a lower MRD on day 42 (*P* = 0.009).

Multivariate logistic regression analysis was performed to detect predictors of high MRD at 15 days (> 1) and 42 days (> 0.01). *ETV6* (exon 1) CNAs were found to be a predictor of a high MRD on day 15 (odds ratio 6.5 (95% CI 1.125–38.003), *P* = 0.037), while a TLC greater than 30,000 was a predictor of a high MRD on day 42 (OR = 4.8 95% CI (1.068–21.772), *P* = 0.041).

**Table 4:** Association between *ETV6* CNAs with MRD response at day 15

Factors	MRD15 ≤ 1 *n* (%)	MRD15 > 1 *n* (%)	*P* value
*ETV6* (exon 1)			0.041
Normal	41 (73.2)	15 (26.8)	
Abnormal	3 (37.5)	5 (62.5)	
*ETV6* (exon 1 A)			0.033
Normal	39 (72.2)	15 (27.8)	
Abnormal	2(28.6)	5 (71.4)	
*ETV6* (exon 2)			0.452
Normal	31 (66.0)	16 (34.0)	
Abnormal	10 (76.9)	3 (23.1)	
*ETV6* (exon 3)			0.912
Normal	37 (68.5)	17 (31.5)	
Abnormal	6 (66.7)	3 (33.3)	
*ETV6* (exon 5)			0.850
Normal	37 (69.8)	16 (30.2)	
Abnormal	6 (66.7)	3 (33.3)	
*ETV6* (exon 8)			1.000
Normal	39 (68.4)	18 (31.6)	
Abnormal	5 (71.4)	2 (28.6)	

### Patient outcomes

**Fig. 2:**
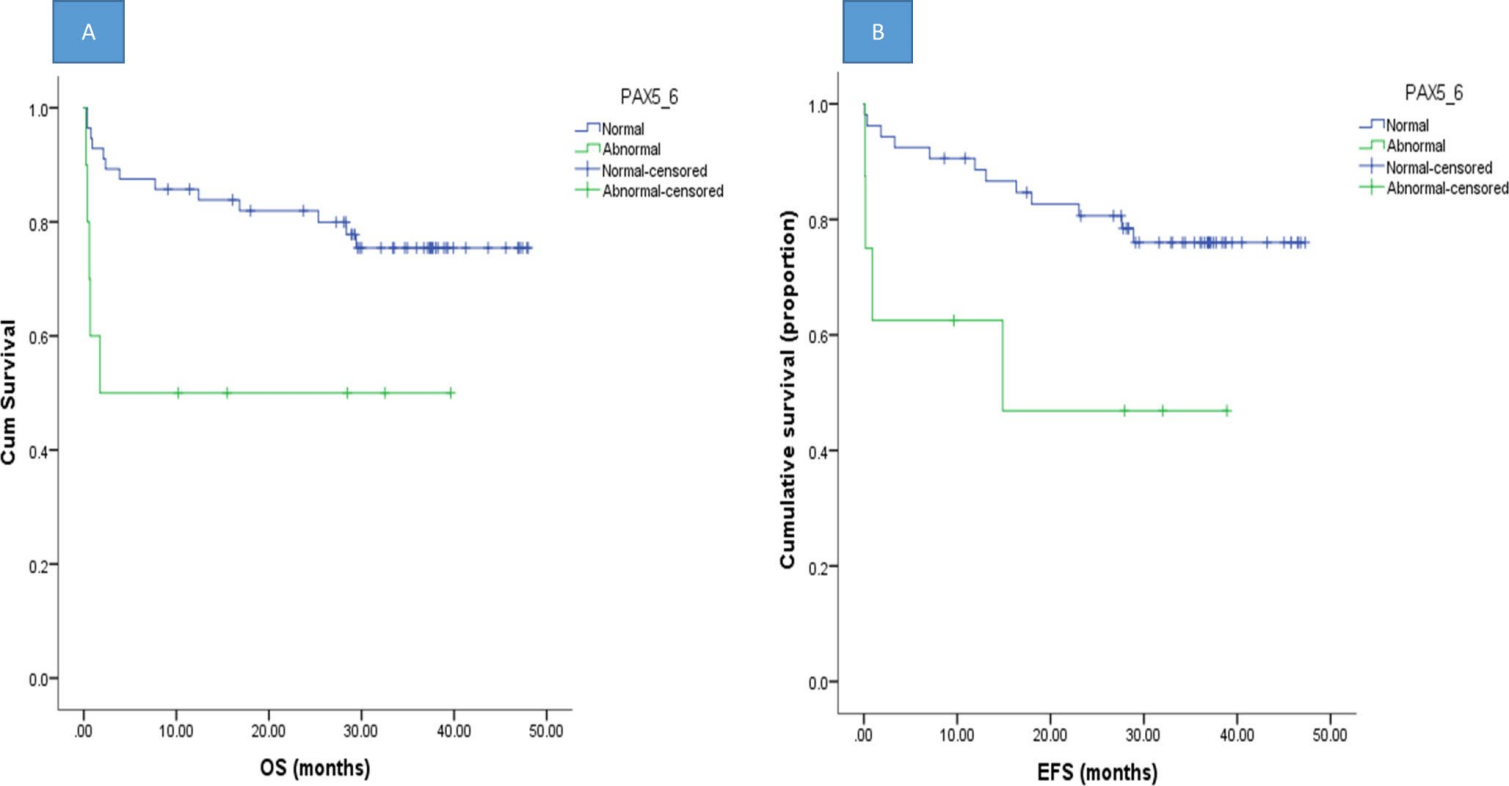
**A**: Association between CNAs of *PAX5* gene and OS, Patients with CNAs in *PAX5* gene (exon 6) had a significantly lower OS (50% versus 85.7%, *P* = 0.012) versus normal copy number respectively **B**: association between *PAX5* CNAs and EFS. Patients with CNAs in *PAX5* (exon 6) had a significantly lower EFS than in those without (62.5% versus 88.6%, *P* = 0.025)

Among the studied 78 patients, number of events was 23, the cumulative OS at 1, 2 and 3years was found to be 77.9%, 75.1%, 68.8% respectively. The association between different demographic, clinical and laboratory data and the patients’ OS were studied, none of them proved to be significant Supplementary Tables (SI 5–6). On the other hand, Cytogenetic risk classification, MRD day 42, NCI risk classification and *PAX5* CNAs were all predictors of OS. Patients at risk of adverse cytogenetic manifestations had significantly shorter OS than did those at intermediate or favorable risk (54.2% vs. 81.5% and 83.9%, respectively; *P* = 0.05). Regarding NCI risk stratification, patients with HRs had shorter OS than those with LRs and SRs (39.2% vs. 80.8% and 89.1%, respectively; *P* = 0.019). Patients with positive MRD on day 42 had a significantly shorter OS than patients with negative MRD (75%, 95%, *P* = 0.012), and MRD on day 15 was not significantly associated with OS ( 5). Patients with *PAX5* CNAs had a significantly shorter OS (50% versus 85.7%, *P* = 0.012) than patients with normal copy number CNAs (Fig. 2). We detected an insignificant association between deletions of the CDKN2A/2B gene (exon 2) or the CNA in the ETV6 gene (exon 1) and hypodiploidy with shorter OS (*P* = 0.08, *P* = 0.09, *P* = 0.054) respectively.

The cumulative EFS at one, two, three years was 84.2%, 76.6%, 71.5% respectively and the number of events was 19. The association between different demographic, clinical and laboratory data and the patients’ EFS were studied, none of them proved to be significant apart from the association between the lower initial Hemoglobin and CD34 expression on blast cells with longer EFS which is on contrary with most previous studies and need further investigation Supplementary Tables (SI 15–16).

The cytogenetics risk and NCI risk stratifications and *PAX5* CNAs were significant predictors of EFS. Patients with adverse cytogenetic risk had significantly shorter EFS than did those with intermediate or favorable cytogenetic risk (44.4% vs. 7.1% and 73.2%, respectively; *P* = 0.031). For the NCI risk classification, patients with HRs had a significantly shorter EFS than patients with SRs and LRs (57.1% vs. 91.3% and 91.2%, respectively; *P* = 0.006) (Table 5). EFS was significantly worse in patients with CNAs in *PAX5* gene exon 6 than in those without CNAs (62.5% versus 88.6%, *P* = 0.025) (Fig. 2). Supplementary Tables (SI 7–14) show the associations between CNAs detected in different experiment genes with patients’ OS and EFS, all these associations were found to be insignificant apart from the association of PAX5 CNAs in certain exons with longer OS and EFS as mentioned before.

**Table 5:** Factors that were significantly associated with shorter Overall survival or Event free

Data	No. of cases	NO. of events	Survival % at 1 year	Survival % at 2 years	Survival % at 3 years	*P* value
*Overall survival*						
Cytogenetic risk						0.050*
Favorable	32	8	83.9	83.9	73.8	
Intermediate	27	5	81.5	81.5	81.5	
Adverse	11	6	54.2	43.6	-	
Numerical abnormalities						0.054
Hypodiploid	15	7	53.3	53.3	53.3	
Diploid	25	7	80.0	75.6	70.2	
Hyper diploid	28	5	88.9	88.9	81.2	
NCI risk stratification						0.019*
SR	37	9	86.1	86.1	73.7	
LR	26	5	80.8	80.8	80.8	
HR and Infantile	14	8	57.1	39.2	39.2	
MRD 15						
≤ 1	48	13	83.3	78.7	70.6	0.436
> 1	21	4	85.7	85.7	81.0	
MRD 42						0.012*
≤ 0.01	41	4	97.6	95.0	89.2	
> 0.01	16	6	81.3	75.0	60.2	
Event free survival						
Cytogenetic risk stratification						
Favorable	30	7	86.7	83.2	76.3	0.031*
Intermediate	24	3	91.7	87.1	87.1	
Adverse	9	5	66.7	44.4	-	
NCI risk stratification						
SR	34	7	91.2	88.2	77.9	0.006*
LR	23	4	91.3	81.4	81.4	
HR and Infantile	12	7	57.1	-	-	

By performing multivariate analysis, lymph node (LN) involvement, cytogenetic risk, NCI risk stratification, MCN, and *PAX5* exon 6 data were analyzed using a Cox regression model, which yielded significance values less than 0.1. The independent data that significantly affected OS were NCI risk stratification and *PAX5* exon 6 status. The hazard ratio of mortality for children classified as being included in the HR and infantile risk groups was 6.2 times greater than that for children in the other risk groups. Abnormal *PAX5* gene expression increased the risk of mortality by 3.8 (95% CI (1.22–11.7), *P* = 0.021) times compared with normal *PAX5* and NCI adverse risk, and infantile PAX5 increased the risk of mortality by 6.2 (95% CI 1.713–22.934, *P* = 0.043).

According to the EFS multivariate analysis, hemoglobin level, cytogenetic risk, CD34, NCI risk stratification, MRD day 15, CNA in *PAX5* gene 5 exon 6 and *ETV6* exon 1 data that had significance values less than 0.1 were added to the Cox regression model to study the independent predictors of EFS. The hazard ratio of recurrence and death for children classified into the HR group and infantile group was 13.96 (95% CI 2.73–71.43), *p* = 0.002) times greater than that for the standard risk group.

### Association between *PAX5* CNAs and *RUNX1* gene abnormalities

A significant association was found between *PAX5* exon 8 CNA and *RUNX1* gene gain (87.5% versus 12.5%, *P* = 0.017), in addition to a positive significant association between *PAX5* exon 10 CNA and *RUNX1* gene translocations (57.1% versus 42.9%, *P* = 0.041), (Table 6).

**Table 6:** Association between *RUNX1* gain and translocation and *PAX5* CNAs

Association between RUNX1 gene gain and PAX5 gene CNAs				Association of RUNX1 with gene translocation PAX5 gene CNAs		
	**YES** **(%)**	**NO** **(%)**	**P value**	**YES** **(%)**	**NO** **(%)**	**P value**
*PAX5* (exon10) (*n* = 67)			0.692			0.041
Normal	26 (43.3)	34 (56.7)		13 (21.7)	47 (78.3)	
Abnormal	4 (57.1)	3 (42.9)		4 (57.1)	3 (42.9)	
*PAX5* (exon 8) (*n* = 63)			0.017			**0.400**
Normal	20 (36.4)	35 (63.6)		14 (25.5)	41 (74.5)	
Abnormal	7 (87.5)	1 (12.5)		3 (42.9)	4 (57.1)	
*PAX5* (exon7) (*n* = 62)			0.119			**0.331**
Normal	21 (38.2)	34 (61.8)		21 (38.2)	34 (61.8)	
Abnormal	5 (71.4)	2 (28.6)		5 (71.4)	2 (28.6)	
*PAX5*(exon 6) (*n* = 66)			0.388			**0.833**
Normal	25 (44.6)	31 (55.4)		15 (26.8)	41 (73.2)	
Abnormal	3 (30.0)	7 (70.0)		3 (30.0)	7 (70.0)	
*PAX5*(exon 5) (*n* = 64)			0.340			**0.700**
Normal	20 (36.2)	31 (60.8)		13 (25.5)	38 (74.5)	
Abnormal	7 (53.8)	6 (46.2)		4 (30.8)	9 (69.2)	
*PAX5*(exon 2) (*n* = 62)			0.953			**0.271**
Normal	23 (43.3)	30 (56.6)		14 (26.4)	39 (73.6)	
Abnormal	4 (44.4)	5 (55.6)		4 (44.4)	5 (55.6)	
*PAX5*(exon 1) (*n* = 67)			0.130			**0.595**
Normal	26 (45.6)	31 (54.4)		16 (28.1)	41 (71.9)	
Abnormal	2 (20.0)	8 (80.0)		2 (20.0)	8 (80.0)	

## Discussion

We aimed to investigate whether CNAs in pediatric BCP-ALL patients can affect the therapeutic response (in the form of MRD) as well as patient outcomes (in the form of OS and EFS). In this study, *ETV6* CNA was significantly associated with E/R translocation (*p* > 0.001). Our results agree with those of previous studies in which deletion of the *ETV6* 12p13 gene was detected in 42–70% of E/R-positive ALL patients [6, 10, 17–19].

In this study, 28.6% of the CNAs in the *ETV6* gene were CNAs, 20.8% of which were heterozygous deletions, 1.4% were homozygous deletions, and 8.4% were heterozygous duplications. A similar frequency of *ETV6* deletions in BCP-ALL patients was reported in previous studies that used SNP-A analysis of BCP-ALL patients and MLPA [20, 21]. Both the *BCL2L14* gene, which encodes a proapoptotic member of the *Bcl-2* family 33, and the *CDKN1* gene, which encodes a cyclin-dependent kinase inhibitor, are additional potential genes affected by deletions at 12p13. Deletions in these genes are linked to short remissions and EFS [6, 9]. In our study, although *ETV6* CNAs were not found to affect patient OS or EFS, *ETV6* CNAs in exons 1 and 1 A were strongly associated with high MRD on day 15, as 62.5% and 71.4% of patients, respectively, had MRD at days 15 > 1 (*P* = 0.041, *P* = 0.033, respectively).

The frequency of CNAs in the *PAX5* gene was 37.1%, of which 28.8% had heterozygous deletions and 11% had heterozygous duplications. A lower frequency of CNAs in the *PAX5* gene was detected in earlier studies [4, 22]. PAX5 gene overexpression occurs in more than 90% of pediatric patients with BCP-ALL. It can fuse with other proteins, such as Janus kinase (Jak) 2, to create an active kinase domain, leading to B-cell proliferation via the *JAK-STAT* signaling pathway [23]. Moreover, the most common recurrent focal deletion region in E/R-positive tumors involves *PAX5* (9p13.2; 25%), and these deletions are thought to be early events in leukemogenesis [24].

We found that 14.5% and 14.1% of the most frequently deleted exons in the *PAX5* gene were exon 2 and exon 5, respectively. The most amplified exon was exon 5, which was previously reported [25]. In our study, deletion of the *PAX5* gene was more common than amplification of this gene (28.8% vs. 11%, respectively). Comparable results were found by [26]. *PAX5* amplification is a defined distinct genetic abnormality. These patients have specific genetic and outcome profiles distinct from those of patients with *PAX5* deletion, including a high frequency of *CDKN2A/B* loss and trisomy 5 [27].

CNAs in the *PAX5* gene were found to be significantly associated with CNAs in other genes, such as *IKZF1* and *CDKN2A/2B* (CI = 1.622–13.249, 1.250–9.800, respectively), and the same co-occurrence was previously described [4, 26].

CNAs are usually cooperative genomic aberrations that correlate with specific genomic subtypes and influence the ultimate patient outcome. Nevertheless, one major limitation in assessing the prognostic relevance of individual CNAs is the fact that many patients harbor more than one deletion, while other patients may have none. Therefore, many attempts have been made to integrate CNA profiles and classifiers into the existing established risk group stratification methods. Ampatzidou et al. used the MLPA assay for isolated CNAs and reported that the gene deletions associated with greater relapse probability were *CDKN2A/2B*,* RB*1 and *IKZF1*, with relapse rates of 41.2%, 25% and 16.7%, respectively, while isolated *ETV6* or *PAX5* gene deletions correlated with no relapse occurrence [22].

In our study, CNAs in *PAX5* were not found to be predictive of high MRD levels on either day 15 or day 42, which is consistent with the findings of previous studies [4]. However, isolated *PAX5* CNAs, especially those in exon 6, were found to be associated with worse OS and EFS (*P* = 0.012, 0.025), which agreed with a study in 2018 that was performed on a group of patients with Philadelphia-negative BCP-ALL [28]. The *PAX5*-plus novel subgroup of BCP-ALL was described by Bastian et al. to harbor a biallelic *PAX5* mutation, p.P80R, as a hotspot with homozygous *CDKN2A/B* deletions and *RAS*-activating hotspot mutations as cooperative events, and a tendency for more favorable clinical outcomes was observed [29].

In a study on an Iranian population by Moafi et al., *PAX5* deletion was the most prevalent CNA observed in 45% of ALL patients and had a significant negative impact on the response to treatment. In this study, Moafi et al. classified patients with deletions in the *PAX5* or *CDKN2A/B* genes or duplications in the *ETV6* gene as high-risk patients and needed more intensified treatment protocols to improve their survival [30]; however, in another study in 2021, *PAX5* CNAs were not found to be an independent predictor of poor outcomes [22].

Gene mutations that predispose to familial leukemia, such as *PAX5*, *TP53*, and *ETV6*, impair function of the encoded protein. Germline *IKZF1* variants associated with leukemia predisposition also directly influence responsiveness of leukemia cells to chemotherapy. These variants affect residues throughout the protein. The majority of germline *IKZF1* variants have deleterious effects on IKAROS function, including impaired DNA binding and regulation of transcriptional targets, induction of aberrant leukemic cell adhesion, and impaired drug responsiveness [31]. In this study, the incidence of CNAs in *IKZF1* was 35.7% (25 patients).

*PAX5* germline variants have been identified in multiple familial B-ALL studies that provided evidence that *PAX5* germline variants can induce B-ALL. Two examples of those *PAX5* germline mutations are heterozygous germline variant *PAX5* G183S, affecting the octapeptide domain of *PAX5* and the *PAX5* R38H germline variant. *PAX5* R38H is associated with an older onset than *PAX5* G183S germline variant, but both shared the feature of disrupting the *PAX5* wild type allele and *CDKN2A/B* genes mostly through 9p deletion. These findings strengthen the conclusion that *PAX5* germline variants can cause B-ALL susceptibility and are associated with specific additional genetic lesions to initiate overt B-ALL [32]. We could not study the germline predisposition of Both *PAX5* and *IKZF1* due to limited budget, however investigating the germline predisposition might lead hidden indications regarding the leukemic transformation in some of our patients.

In this study, *RUNX1* gene abnormalities were studied using a break-apart FISH probe to detect *RUNX1* gene amplification, deletion, and translocation. In a previous study, Abbasi et al. assessed genome-wide large and fine-scale CNAs and identified chromosome 21-associated CNAs in 258 patients (49%). Four distinct types of CNAs were found: direct fusion-related intragenic *RUNX1* deletions, duplication of the non-rearranged homolog, and duplications of either the distal or the proximal translocated parts in the form of a der (12) or a der (21), respectively [33]. Interestingly, we found a significant association between *PAX5* CNAs and *RUNX1* gain and translocation (*P* = 0.017 and *P* = 0.041, respectively), which was in line with previous studies reporting the same association between *PAX5* CNAs and ETV6::RUNX1 translocation [18, 25].

Little is known about the impact of TLC at diagnosis on MRD levels and patient outcomes. Akahoshi et al. evaluated adult patients with Ph^+^ ALL who achieved negative MRD and received HSCT in first complete remission using a threshold of 15,000/µL TLC at diagnosis and reported that high TLC was associated with an increased risk of hematological relapse [34]. In our study, using a cutoff of 30,000/µL, patients with a low initial TLC had significantly lower MRD on day 42 (*P* = 0.009).

## Conclusion

*ETV6* CNAs were significantly associated with E/R translocation. *ETV6* CNAs in exons 1 and 1 A were strongly associated with high MRD on day 15. CNAs in the *PAX5* gene were found to be significantly associated with CNAs in other genes, such as *IKZF1* and *CDKN2A/2B.* Isolated *PAX5* CNAs, especially those in exon 6, were found to be associated with worse OS and EFS. A significant association between *PAX5* CNAs and *RUNX1* gain, and translocation was found. Patients with a low initial TLC had significantly lower MRD on day 42.

## Electronic supplementary material

Below is the link to the electronic supplementary material.

Supplementary Material 1

## Data Availability

No datasets were generated or analysed during the current study.

## Abbreviations

ANC: Absolute neutrophilic count
ALL: Acute lymphoblastic leukemia
BCP-ALL: B-cell precursor ALL
BM: Bone marrow
CNS: Central nervous system
CR: Complete remission
CI: Confidence Interval
CAN: Copy-number alterations
E/R: ETV6::RUNX1
EFS: Event-free survival
FR: Final ratio
FISH: Fluorescence in situ hybridization
HR: High risk
HLA: Human leucocytic antigen
IPT: Immunophenotyping
IRB: Institutional Review Board
iAMP21: Intrachromosomal amplification of chromosome 21
Jak: Janus kinase
LR: Low risk
LN: Lymph nodes
MRD: Minimal residual disease
MRD: Minimal residual disease
MCN: Modal chromosome number
MLPA: Multiplex Ligation-dependent Probe Amplification
NCI: National Cancer Institute
OR: Odds Ratio
OS: Overall survival
OS: Overall survival
PB: Peripheral blood
PCR: Polymerase chain reaction
RT‒PCR: Reverse transcription polymerase chain reaction
SR: Standard risk
SPSS: Statistical Package for Social Science
TLC: Total leucocytic count
TXV: Total XV protocol
