# Supplementary material for: Copy number alterations in pediatric B-cell precursor acute lymphoblastic leukemia patients and their association with patients’ outcome
Source: Ann Hematol. 2024 Nov 26;104(3):1821–32. doi: 10.1007/s00277-024-06102-2 (PMC12031935; doi:10.1007/s00277-024-06102-2)
Supplement: Supplementary file 1 — Supplementary Material 1 [file 277_2024_6102_MOESM1_ESM.pdf]

Copy number alterations in pediatric B-cell precursor acute lymphoblastic leukemia patients and their association with patients' outcome

Nesma E. Abdelfattah[1], Ghada M. Elsayed[1], Amira H. Soliman[1], Emad N. Ebeid [2], Mona S. El Ashry[1]

1 Clinical Pathology Department, National Cancer Institute, Cairo University, Cairo, Egypt

2 Pediatric Oncology Department, National Cancer Institute, Cairo University, Cairo, Egypt

Running title: CNA in pediatric BCP-ALL

Corresponding author: Nesma E. Abdelfattah

Clinical Pathology Department, National Cancer Institute, Cairo University, Cairo, Egypt.

Address: Kasr Al Eini Street, 4 Fom El Khalig, Cairo, Egypt.

Postal code: 11796

Telephone: +2-011-11533760

Fax: +202 2365 4880

Email: Nesmaelsayed@cu.edu.eg

ORCID: 0009-0003-2893-0744

A- Supplementary tables

SI (1): CNAs in different exons of *EBF1*and *IKZF1* genes.

Gene/exon	Total	Normal copy number	Heterozygous deletion	Heterozygous duplication	Complete deletion
***EBF1* gene, 5q33.3**					
(*EBF1*-16)	67	61(91.0)	4(6.0)	2(3.0)	0
(*EBF1*-14)	62	59(95.2)	2(3.2)	1(1.6)	0
(*EBF1*-10)	65	56(86.2)	4(6.2)	5(7.7)	0
(*EBF1*-1)	64	58(90.6)	3(4.7)	3(4.7)	0
***IKZF1 gene*, 7p12.2**					
(*IKZF1*-1)	65	57(87.6)	7(10.8)	1(1.5)	0
(*IKZF1*-2)	65	57(87.7)	5(7.7)	3(4.6)	0
(*IKZF1*-3)	67	53(67.9)	5(7.5)	9(13.4)	0
(*IKZF1*-4)	67	54(80.6)	13(19.4)		0
(*IKZF1*-5)	67	51(75.0)	14(20.6)	1(1.5)	0
(*IKZF1*-6)	65	46(70.7)	12(18.5)	7(10.8)	0
(*IKZF1*-7)	63	51(79.8)	11(17.2)	1(1.6)	0
(*IKZF1*-8)	65	60(92.3)	5(7.7)		0

Data are presented as numbers (percentages) unless otherwise indicated

SI (2): CNA in different exons of *CDKN2A/CDKN2B* and *PAX5* genes.

Gene/exon	Total	Normal copy number	Heterozygous deletion	Heterozygous duplication	Complete deletion
CDKN2A/CDKN2B					
(JAK2-23)	68	59(86.8)	6(8.8)	3(4.4)	0
(CDKN2A-4)	64	45(70.3)	17(26.6)	2(3.1)	0
(CDKN2A-2)	63	48(76.2)	15(23.8)	0	0
(CDKN2B-2)	65	44(67.7)	18(27.7)	2(3.1)	1(1.5)
PAX5 gene, 9p13.2 (n=67)					
(PAX5 -10)	67	60(89.6)	6(8.9)	1(1.5)	0
(PAX5 -8)	63	55(87.3)	5(7.9)	3(4.8)	0
(PAX5 -7)	62	55(88.7)	7(11.3)	0	0
(PAX5 -6)	66	56(84.8)	8(12.1)	2(3.0)	0
(PAX5 -5)	64	51(79.7)	9(14.1)	4(6.3)	0
(PAX5 -2)	62	53(85.5)	9(14.5)	0	0
(PAX5 -1)	67	57(85.1)	7(10.4)	3(4.5)	0

Data are presented as numbers (percentages) unless otherwise indicated

SI (3): CNA in different exons of *ETV6 and BTG1*gene.

Gene/exon	Total	Normal copy number	Heterozygous deletion	Heterozygous duplication	Complete deletion
ETV6 gene, 12p13.2					
(ETV6 -1)	70	62(88.6)	2(2.9)	5(7.1)	1(1.4)
(ETV6 -1A)	67	60(89.6)	3(4.5)	4(6.0)	0
(ETV6 -2)	66	53(80.3)	10(15.2)	3(4.5)	0
(ETV6 -3)	69	60(87.0)	7(10.1)	2(2.9)	0
(ETV6 -5)	68	58(85.3)	7(10.3)	3(4.4)	0
(ETV6 -8)	70	62(88.6)	5(7.1)	3(4.3)	0
BTG1 gene, 12q21.33					
(BTG1-AREA-down)	70	68(97.1)	0	2(2.9)	0
(BTG1-AREA-down-A)	68	63(92.6)	4(5.9)	1(1.5)	0
(BTG1-2)	69	62(89.9)	4(5.8)	3(4.3)	0
(BTG1-1)	68	62(91.2)	1(1.5)	5(7.3)	0

Data are presented as numbers (percentages) unless otherwise indicated

SI (4): CNAs in different exons of *RB1* gene *and Xp22.33 / Yp11.32 region.*

Gene/exon	Total	Normal copy number	Heterozygous deletion	Heterozygous duplication	Complete deletion
RB1 gene, 13q14.2					
(RB1-6)	68	67(98.5)	0	1(1.5)	0
(RB1-14)	67	65(97.0)	0	2(3.0)	0
(RB1-19)	68	67(98.5)	1(1.5)	0	0
(RB1-24)	69	68(98.6)	1(1.4)	0	0
(RB1-26)	69	65(94.2)	3(4.3)	1(1.4)	0
Xp22.33 / Yp11.32 region					
SHOX-AREA-down	63	50(79.4)	0	13(20.6)	0
CRLF2-4	65	49(75.4)	0	16(24.6)	0
CSF2R4-10	66	52(78.8)	0	14(21.2)	0
IL3RA-1	64	48(75.0)	1(1.6)	15(23.4)	0
P2RY8-2	66	44(66.7)	1(1.5)	21(31.8)	0
ZFT-4(YC)	66	60(90.9)	0	3(4.5)	3 (4.5)

Data are presented as numbers (percentages) unless otherwise indicated

SI (5): Association between demographic and clinical variables and Overall survival

Data	Number. of cases	Number. of events	Cumulative survival at 1 year (%)	Cumulative survival at 2 year (%)	Cumulative survival at 3 year (%)	Median survival time (months)	*P* value
**Whole group**	78	23	77.9	75.1	68.8	NR	
**Age (n=74)**							
**1-9 years**	55	13	81.5	81.5	74.4	NR	0.58
**≥ 10years**	19	6	78.3	73.3	67.6	NR	
**Sex**							
**Male**	47	13	84.8	80.0	69.1	NR	0.60
**Female**	31	10	67.7	67.7	67.7	NR	
**Hepatomegaly**							
**Positive**	23	7	78.2	74.5	70.4	NR	0.80
**Negative**	55	16	77.3	77.3	64.8	NR	
**Splenomegaly**							
**Positive**	59	19	76.3	72.7	66.7	NR	0.49
**Negative**	19	4	83.3	83.3	76.4	NR	
**LN**							
**Positive**	51	12	82.4	82.4	74.8	NR	0.09
**Negative**	27	11	69.2	61.5	57.4	NR	
**Initial CNS affection**							
**Positive**	6	3	50.0	50.0	-	2.35	0.19
**Negative**	72	20	80.3	77.2	70.4	NR	
**Fever**							
**Positive**	54	16	77.8	75.7	69.1	NR	0.98
**Negative**	24	7	78.3	73.9	68.2	NR	
**Anemia**							
**Positive**	68	19	77.6	77.6	70.5	NR	0.46
**Negative**	10	4	80.0	58.3	58.3	NR	
**Bleeding**							
**Positive**	30	11	70.0	66.3	62.6	NR	0.19
**Negative**	48	12	83.0	80.7	72.7	NR	

NR: not reached

SI (6): Association between different laboratory data and OS

Data	Number of cases	Number of events	Survival % at 1 year	Survival % at 2 year	Survival % at 3 year	Median survival time (months)	*P* value
**Total leucocytic count (×10^9^/L)**							
**≤ 30**	34	8	79.4	79.4	75.8	NR	0.330
**>30**	44	15	76.2	71.3	62.6	NR	
**Hemoglobin (gm/dl)**							
**≤ 11 gm/dl**	73	20	77.8	77.8	71.1	NR	0.158
**>11 gm/dl**	5	3	80.0	-	-	16.8	
**Platelet (×10^9^/L)**							
**≤ 100**	64	18	77.8	76.1	70.5	NR	0.733
**>100**	14	5	78.6	71.4	62.5	NR	
**IPT diagnosis**							
**PreB**	41	11	77.5	77.5	72.3	NR	
**ProB**	4	2	75.0	-	-	NR	NA
**CommonALL**	33	10	78.8	75.2	67.1	NR	
**CD34**							
**Positive**	40	14	87.5	82.4	76.6	NR	0.103
**Negative**	38	9	67.6	67.6	60.4	NR	
**Aberrant myeloid marker**							
**Positive**	6	2	83.3	66.7	-	NR	0.970
**Negative**	72	21	77.5	76.0	69.0	NR	
**DNA Index**							
**≤ 1**	58	18	75.9	72.2	67.6	NR	0.611
**> 1**	20	5	84.2	78.2`1	72.2	NR	

NR: not reached

SI (7): Association of EBF1 gene CNAs with EFS and OS

Factors	Event free Survival						Overall- Survival					
	No. (70)	No. of events	Cumulative Survival at			P value	No. (78)	No. of events	Cumulative Survival			P value
			1 year (%)	2 year (%)	3year (%)				1 year (%)	2 year (%)	3 year (%)	
***EBF1* gene, 5q33.3 (*EBF1*-16) (Combined categories) (N=62)**							(n=67)					
**Normal**	56	14	83.9	76.4	74.3	0.619	61	16	78.7	75.2	73.2	0.554
**Abnormal**	6	1	80.0	80.0	-		6	1	80.0	80.0	-	
***EBF1* gene, 5q33.3 (*EBF1*-16) (N=62)**							(n=67)					
**Equal**	56	14	83.9	76.4	74.3	NA	61	16	78.7	75.2	73.2	NA
**Heterozygous deletion**	4	0	-	-	-		4	0	-	-	-	
**Heterozygous duplication**	2	1	-	-	-		2	1	-	-	-	
***EBF1* gene, 5q33.3 (*EBF1*-14) (Combined categories) (N=57)**							(n=62)					
**Normal**	59	16	83.2	75.5	73.3	NA	59	16	78.0	74.3	72.2	NA
**Abnormal**	3	1	-	-	-		3	1	50.0	50.0	50.0	
***EBF1* gene, 5q33.3 (*EBF1*-14) (N=57)**							(n=62)					
**Equal**	54	16	83.2	75.5	73.3	NA	59	16	78.0	74.3	72.2	
**Heterozygous deletion**	2	0	-	-	-		2	0	-	-	-	NA
**Heterozygous duplication**	1	1	-	-	-		1	1	-	-	-	
***EBF1* gene, 5q33.3 (*EBF1*-10)(Combined categories) (N=60)**							(n=65)					
**Normal**	51	13	84.2	75.9	73.6	0.784	56	15	78.6	74.7	72.5	0.669
**Abnormal**	9	2	88.9	88.9	76.2		9	2	88.9	76.2	76.2	
***EBF1* gene, 5q33.3 (*EBF1*-10) (N=60)**							(n=65)					
**Equal**	51	13	84.2	75.9	73.6	NA	56	15	78.6	74.7	72.5	NA
**Heterozygous deletion**	4	0	-	-	-		4	0	-	-	-	
**Heterozygous duplication**	5	2	80.0	80.0	-		5	2	80.0	80.0	60.0	
***EBF1* gene, 5q33.3 (*EBF1*-1) (Combined categories) (N=59)**							(n=64)					
**Normal**	53	14	82.9	75.0	72.8	0.572	58	16	77.6	73.9	71.7	0.512
**Abnormal**	6	1	100.0	100.0	-		6	1	80.0	80.0	-	
***EBF1* gene, 5q33.3 (*EBF1*-1) (N=59)**							(n=64)					
**Equal**	53	14	82.9	75.0	72.8	NA	58	15	77.6	73.9	71.7	NA
**Heterozygous deletion**	3	0	-	-	-		3	0	-	-	-	

NA: Not applicable

SI (8): Association of *IKZF1*gene CNAs with EFS and OS

**Factors**	**Event Free- Survival (EFS)**						**Overall- Survival (OS)**					
	**No. (70)**	**No. of events**	**Cumulative Survival at**			**P value**	**No. (78)**	**No. of events**	**Cumulative Survival**			***P* value**
			**1 year (%)**	**2 year (%)**	**3year (%)**				**1 year (%)**	**2year (%)**	**3 year (%)**	
**Heterozygous deletion**	12	3	83.3	75.0	75.0		13	3	76.9	76.9	76.9	
***IKZF1* gene, 7p12.2 (*IKZF1*-5) (N=61) (Combined categories)**												
**Normal**	47	12	84.9	78.0	72.4	NA	58	16	77.6	73.9	71.7	0.512
**Abnormal**	14	3	85.7	78.6	78.6		6	1	80.0	80.0	-	
***IKZF1* gene, 7p12.2 (*IKZF1*-5) (N=61)**												
**Equal**	47	12	84.9	78.0	72.4	NA	58	15	77.6	73.9	71.7	NA
**Heterozygous deletion**	13	3	84.6	76.9	76.9		3	0	66.7	66.7	-	
**Heterozygous duplication**	1	0	-	-	-		3	1	-	-	-	
***IKZF1* gene, 7p12.2 (*IKZF1*-6) (N=60) (Combined categories)**												
**Normal**	42	10	88.1	80.5	74.6	NA	46	13	80.4	78.1	70.1	0.933
**Abnormal**	18	5	77.4	71.4	71.4		19	5	78.9	73.3	73.3	
***IKZF1* gene, 7p12.2 (*IKZF1*-6) (N=60)**												
**Equal**	42	10	88.1	80.5	74.6	NA	46	12	80.4	78.1	70.1	0.984
**Heterozygous deletion**	11	3	81.8	72.7	72.7		12	3	75.0	75.0	75.0	
**Heterozygous duplication**	7	2	68.6	68.6	-		7	2	68.6	68.6	-	
***IKZF1* gene, 7p12.2 (*IKZF1*-7) (N=58) (Combined categories)**												
**Normal**	88.1	80.5	74.6	88.1	80.5	NA	51	14	80.4	78.2	70.8	0.918
**Abnormal**	11	3	81.8	72.7	72.7		12	3	75.0	75.0	75.0	
***IKZF1* gene, 7p12.2 (*IKZF1*-7) (N=58)**												
**Equal**	47	11	87.2	80.3	74.8	NA	51	14	80.4	78.2	70.8	NA
**Heterozygous deletion**	10	3	80.0	70.0	70.0		11	3	72.7	72.7	72.7	
**Heterozygous duplication**	1	0	-	-	-		1	0	-	-	-	
***IKZF1* gene, 7p12.2 (*IKZF1*-8) (N=60)**												
**Equal**	56	15	85.7	76.1	71.5	NA	60	16	80.0	78.1	71.7	0.762
**Heterozygous deletion**	4	0					5	1	80.0	80.0	-	

NA: Not applicable

SI (8): Continued

Factors	Event free Survival						Overall- Survival					
	No. (70)	No. of events	Cumulative Survival at			P value	No. (78)	No. of events	Cumulative Survival			P value
			1 year (%)	2 year (%)	3year (%)				1 year (%)	2 year (%)	3 year (%)	
**Heterozygous deletion**	12	3	83.3	75.0	75.0		13	3	76.9	76.9	76.9	
***IKZF1* gene, 7p12.2 (*IKZF1*-5) (n=61) (Combined categories)**												
**Normal**	47	12	84.9	78.0	72.4	0.33	58	16	77.6	73.9	71.7	0.512
**Abnormal**	14	3	85.7	78.6	78.6		6	1	80.0	80.0	-	
***IKZF1* gene, 7p12.2 (*IKZF1*-5) (n=61)**												
**Equal**	47	12	84.9	78.0	72.4	0.43	58	15	77.6	73.9	71.7	NA
**Heterozygous deletion**	13	3	84.6	76.9	76.9		3	0	66.7	66.7	-	
**Heterozygous duplication**	1	0	-	-	-		3	1	-	-	-	
***IKZF1* gene, 7p12.2 (*IKZF1*-6) (n=60) (Combined categories)**												
**Normal**	42	10	88.1	80.5	74.6	0.45	46	13	80.4	78.1	70.1	0.933
**Abnormal**	18	5	77.4	71.4	71.4		19	5	78.9	73.3	73.3	
***IKZF1* gene, 7p12.2 (*IKZF1*-6) (n=60)**												
**Equal**	42	10	88.1	80.5	74.6	0.72	46	12	80.4	78.1	70.1	0.984
**Heterozygous deletion**	11	3	81.8	72.7	72.7		12	3	75.0	75.0	75.0	
**Heterozygous duplication**	7	2	68.6	68.6	-		7	2	68.6	68.6	-	
***IKZF1* gene, 7p12.2 (*IKZF1*-7) (n=58) (Combined categories)**												
**Normal**	88.1	80.5	74.6	88.1	80.5	0.45	51	14	80.4	78.2	70.8	0.918
**Abnormal**	11	3	81.8	72.7	72.7		12	3	75.0	75.0	75.0	
***IKZF1* gene, 7p12.2 (*IKZF1*-7) (n=58)**												
**Equal**	47	11	87.2	80.3	74.8	0.5	51	14	80.4	78.2	70.8	NA
**Heterozygous deletion**	10	3	80.0	70.0	70.0		11	3	72.7	72.7	72.7	
**Heterozygous duplication**	1	0	-	-	-		1	0	-	-	-	
***IKZF1* gene, 7p12.2 (*IKZF1*-8) (n=60)**												
**Equal**	56	15	85.7	76.1	71.5	NA	60	16	80.0	78.1	71.7	0.762
**Heterozygous deletion**	4	0					5	1	80.0	80.0	-	

NA: Not applicable

SI (9): Association of *CDKN2A/CDKN2B* gene CNAs with EFS and OS

Factors	Event free Survival						Overall- Survival					
	No. (70)	No. of events	Cumulative Survival at			P value	No. (78)	No. of events	Cumulative Survival			P value
			1 year (%)	2 year (%)	3year (%)				1 year (%)	2 year (%)	3 year (%)	
***CDKN2A/CDKN2B*, 9p21.3 (JAK2-23) (n=63) (Combined categories)**							**n =68**					
**Normal**	54	14	85.1	77.5	73.3	0.455	59	17	79.7	76.1	70.1	0.319
**Abnormal**	9	1	88.9	88.9	-		9	1	88.9	88.9	-	
***CDKN2A/CDKN2B*, 9p21.3 (JAK2-23) (n=63)**							**n=68**					
**Equal**	54	14	85.1	77.5	73.3	NA	59	17	79.7	76.1	70.1	NA
**Heterozygous deletion**	6	1	-	-	-		6	1	83.3	-	-	
**Heterozygous duplication**	3	0	-	-	-		3	0	-	-	-	
***CDKN2A/CDKN2B*, 9p21.3 (CDKN2A4) (n=59) (Combined categories)**							**n=64**					
**Normal**	42	11	85.7	78.5	73.3	0.680	45	13	82.2	77.7	70.3	0.748
**Abnormal**	17	3	88.2	80.9	-		19	4	78.9	78.9	78.9	
***CDKN2A/CDKN2B*, 9p21.3 (CDKN2A4) (n=59)**							**n=64**					
**Equal**	42	11	85.7	78.5	73.3	NA	45	13	82.2	77.7	70.3	NA
**Heterozygous deletion**	16	3	87.5	79.5	-		17	3	82.4	82.4	82.4	
**Heterozygous duplication**	1	0	-	-	-		2	1	-	-	-	
***CDKN2A/CDKN2B*, 9p21.3 (CDKN2A2) (n=56)**							**n=63**					
**Equal**	44	11	88.5	78.8	72.7	0.448	48	12	85.1	80.6	72.2	0.088
**Heterozygous deletion**	12	4	66.7	66.7	-		15	7	53.3	53.3	53.3	
***CDKN2A/CDKN2B*, 9p21.3 (CDKN2B2) (n=60) (Combined categories)**							**n=65**					
**Normal**	40	11	82.5	74.9	72.0	0.380	44	14	77.3	72.7	67.5	0.190
**Abnormal**	21	3	90.0	84.0	84.0		21	3	85.7	85.7	85.7	
***CDKN2A/CDKN2B*, 9p21.3 *CDKN2A/CDKN2B*, 9p21.3 (CDKN2B2) (n=60)**							**n=65**					
**Equal**	40	11	82.5	74.9	72.0	NA	44	15	77.3	72.7	67.5	NA
**Heterozygous deletion**	17	3	88.2	80.9	-		18	0	83.3	83.3	83.3	
**Heterozygous duplication**	2	0	-	-	-		2	1	-	-	-	
**Complete deletion**	1	0	-	-	-		1	0	-	-	-	

NA: Not applicable

SI (10): Association of *PAX5* gene CNAs with EFS and OS

Factors	Event free Survival						Overall- Survival					
	No. (70)	No. of events	Cumulative Survival			P value	No. (78)	No. of events	Cumulative Survival			P value
			1 year (%)	2year (%)	3year (%)				1year (%)	2year (%)	3year (%)	
***PAX5* gene, 9p13.2 (*PAX5* -10) (n=62) (Combined categories)**							**n=67**					
**Normal**	55	14	83.5	75.7	73.3	0.468	60	16	78.3	74.7	72.4	0.427
**Abnormal**	7	1	85.7	85.7	-		7	1	85.7	85.7	-	
***PAX5* gene, 9p13.2 (*PAX5* -10) (n=62)**							**n=67**					
**Equal**	55	14	83.5	75.7	73.3	NA	60	16	78.3	74.7	72.4	NA
**Heterozygous deletion**	6	1	83.3	83.3	-		6	1	83.3	83.3	-	
**Heterozygous duplication**	1	0	-	-	-		1	0	-	-	-	
***PAX5* gene, 9p13.2 (*PAX5* -8) (n=58) (Combined categories)**							**n=63**					
**Normal**	51	15	82.2	73.8	68.9	0.394	55	16	78.2	74.2	69.3	0.817
**Abnormal**	7	1	83.3	83.3	-		8	2	87.5	87.5	-	
***PAX5* gene, 9p13.2 (*PAX5* -8) (n=58)**							**n=63**					
**Equal**	51	15	82.2	73.8	68.9	NA	55	16	78.2	74.2	69.3	NA
**Heterozygous deletion**	5	1	75.0	75.0	-		5	1	100.0	75.0	-	
**Heterozygous duplication**	2	0	66.7	-	-		3	1	66.7	-	-	
***PAX5* gene, 9p13.2 (*PAX5* -7) (n=57)**							**n=62**					
**Equal**	50	13	83.8	77.4	72.3	0.943	55	16	78.2	74.2	69.3	0.884
**Heterozygous deletion**	7	2	85.7	71.4	-		7	2	85.7	85.7	-	
***PAX5* gene, 9p13.2 (*PAX5* -6) (n=61) (Combined categories)**							**n=66**					
**Normal**	53	12	88.6	80.6	76.0	0.025	56	13	85.7	81.9	75.4	0.012
**Abnormal**	8	4	62.5	-	-		10	5	50.0	-	-	
***PAX5* gene, 9p13.2 (*PAX5* -6) (n=61)**							**n=66**					
**Equal**	53	12	88.6	80.6	76.0	NA	56	13	85.7	81.9	75.4	NA
**Heterozygous deletion**	7	3	71.4	-	-		8	3	-	-	-	
**Heterozygous duplication**	1	1	-	-	-		2	2	-	-	-	
***PAX5* gene, 9p13.2 (*PAX5* -5) (n=5) (Combined categories)**							**n=64**					
**Normal**	47	11	89.4	80.3	74.9	0.126	51	13	82.4	80.2	72.8	0.280
**Abnormal**	12	5	65.6	56.3	56.3		13	5	69.2	60.6	60.6	

NA: Not applicable

SI (10): Continued

Factors	Event free Survival						Overall- Survival					
	No. (70)	No. of events	Cumulative Survival			P value	No. (78)	No. of events	Cumulative Survival			P value
			1 year (%)	2year (%)	3year (%)				1 year (%)	2year (%)	3year (%)	
***PAX5* gene, 9p13.2 (*PAX5* -5) (n=59)**							**n=64**					
**Equal**	47	11	89.4	80.3	74.9	NA	51	13	82.4	80.2	72.8	NA
**Heterozygous deletion**	8	4	62.5	-	-		9	4	55.6	-	-	
**Heterozygous duplication**	4	1	-	-	-		4	1	-	-	-	
***PAX5* gene, 9p13.2 (*PAX5* -2) (n=57)**							**n=62**					
**Equal**	49	10	89.7	83.2	78.2	0.160	53	13	84.9	80.9	73.9	0.933
**Heterozygous deletion**	8	3	62.5	62.5	-		9	4	55.6	55.6	-	
***PAX5* gene, 9p13.2 (*PAX5* -1) (n=62) (Combined categories)**							**n=67**					
**Normal**	52	12	84.5	80.5	75.6	0.841	57	17	78.9	75.3	69.0	0.228
**Abnormal**	10	2	90.0	78.8	78.8		12	3	90.0	90.0	90.0	
***PAX5* gene, 9p13.2 (*PAX5* -1) (n=62)**							**n=67**					
**Equal**	52	12	84.5	80.5	75.6	NA	57	17	78.9	75.3	69.0	NA
**Heterozygous deletion**	7	0	100.0	100.0	-		7	0	100.0	100.0	-	
**Heterozygous duplication**	3	2	-	-	-		3	1	-	-	-	

NA: Not applicable

SI (11): Association of *ETV6* gene CNAs with EFS and OS

Factors	Event free Survival						Overall- Survival					
	No. (70)	No. of events	Cumulative Survival			P value	No. (78)	No. of events	Cumulative survival			P value
			1 year (%)	2 year (%)	3year (%)				1 year (%)	2 year (%)	3 year (%)	
***ETV6* gene, 12p13.2 (*ETV6* -1) (n=65) (Combined categories)**							**n=70**					
**Normal**	57	16	84.1	74.7	70.2	0.097	62	18	79.0	75.5	69.3	0.090
**Abnormal**	8	0	100.0	100.0	100.0		8	0	100.0	100.	-	
***ETV6* gene, 12p13.2 (*ETV6* -1) (n=65)**							**n=70**					
**Equal**	57	16	84.1	74.7	70.2	NA	62	18	79.0	75.5	69.3	NA
**Heterozygous deletion**	2	0	100.0	100.0	-		2	0	83.3	83.3	-	
**Heterozygous duplication**	5	0	-	-	-		5	0	-	-	-	
**Complete deletion**	1	0	-	-	-		1	0	-	-	-	
***ETV6* gene, 12p13.2 (*ETV6* -1_A) (n=62) (Combined categories)**							**n=67**					
**Normal**	55	15	83.5	75.6	71.0	0.127	60	18	78.3	74.7	68.5	0.107
**Abnormal**	7	0	100.0	100.0	100.0		7	0	100	100	100	
***ETV6* gene, 12p13.2 (*ETV6* -1_A) (n=62)**							**n=67**					
**Equal**	55	15	83.5	75.6	71.0	NA	60	18	78.3	74.7	68.5	NA
**Heterozygous deletion**	3	0	-	-	-		3	0	-	-	-	
**Heterozygous duplication**	4	0	-	-	-		4	0	-	-	-	
***ETV6* gene, 12p13.2 (*ETV6* -2) (n=61) (Combined categories)**							**n=66**					
**Normal**	48	11	83.2	76.4	76.4	0.736	53	14	77.4	73.2	73.2	0.681
**Abnormal**	13	4	84.6	76.9	69.2		13	3	92.3	92.3	75.5	
***ETV6* gene, 12p13.2 (*ETV6* -2) (n=61)**							**n=66**					
**Equal**	48	11	83.2	76.4	76.4	NA	53	14	77.4	73.2	73.2	NA
**Heterozygous deletion**	10	3	90.0	80.0	70.0		10	3	90.0	90.0	70.0	
**Heterozygous duplication**	3	1	-	-	-		3	0	-	-	-	
***ETV6* gene, 12p13.2 (*ETV6* -3) (n=64) (Combined categories)**							**n=69**					
**Normal**	55	15	83.5	75.7	71.0	0.262	60	17	78.3	74.7	70.1	0.241
**Abnormal**	9	1	100.0	88.9	88.9		9	1	88.9	88.9	88.9	

NA: Not applicable

SI (11): Continued

Factors	Event free Survival						Overall- Survival					
	No. (70)	No. of events	Cumulative Survival at			P value	No. (78)	No. of events	Cumulative Survival			P value
			1 year (%)	2 year (%)	3year (%)				1 year (%)	2 year (%)	3 year (%)	
***ETV6* gene, 12p13.2 (*ETV6* -3) (n=64)**							**n=69**					
Equal	55	15	83.5	75.7	71.0	NA	60	17	78.3	74.7	70.1	NA
Heterozygous deletion	7	1	100.0	85.7	85.7		7	1	85.7	85.7	85.7	
Heterozygous duplication	2	0	-	-	-		2	0	-	-	-	
***ETV6* gene, 12p13.2 (*ETV6* -5) (n=63) (Combined categories)**							**n=68**					
Normal	54	14	83.2	77.2	72.4	0.689	58	16	79.3	75.6	71.0	0.596
Abnormal	9	2	100.0	77.8	77.8		10	2	90.0	78.8	78.8	
***ETV6* gene, 12p13.2 (*ETV6* -5) (n=63)**							**n=68**					
Equal	54	14	83.2	77.2	72.4	NA	58	16	79.3	75.6	71.0	NA
Heterozygous deletion	6	1	100.0	83.3	83.3		7	2	85.7	85.7	71.4	
Heterozygous duplication	3	1	-	-	-		3	0	-	-	-	
***ETV6* gene, 12p13.2 (*ETV6* -8) (n=65) (Combined categories)**							**n=70**					
Normal	58	15	84.4`	77.0	72.6	0.439	62	16	80.6	77.1	72.8	0.895
Abnormal	7	1	87.5	75.0	75.0		8	2	87.5	75.0	75.0	
***ETV6* gene, 12p13.2 (*ETV6* -8) (n=65)**							**n=70**					
Equal	58	15	84.4`	77.0	72.6	NA	62	16	80.6	77.1	72.8	NA
Heterozygous deletion	5	1	100.0	85.7	85.7		5	1	100	100	80.0	
Heterozygous duplication	2	0	-	-	-		3	1	-	-	-	

SI (12): Association of *BTG1* gene CNAs with EFS and OS

Factors	Event free Survival						Overall- Survival					
	No. (70)	No. of events	Cumulative Survival at			P value	No. (78)	No. of events	Cumulative Survival			P value
			1 year (%)	2 year (%)	3year (%)				1 year (%)	2 year (%)	3 year (%)	
***BTG1* gene, 12q21.33 (*BTG1*-AREA-down) (N=65)**							**n=70**					
**Equal**	68	16	85.6	77.2	73.2	NA	68	18	80.9	77.7	72.2	NA
**Heterozygous duplication**	2	0	-	-	-		2	0	-	-	-	
***BTG1* gene, 12q21.33 (*BTG1*-AREA-down_A) (N=63) (Combined categories)**							n=68					
**Normal**	58	13	87.8	78.6	76.3	0.301	63	15	82.5	79.1	75.1	0.417
**Abnormal**	5	2	-	-	-		5	2	60.0	60.0	60.0	
***BTG1* gene, 12q21.33 (*BTG1*-AREA-down_A) (N=63)**							**n=68**					
**Equal**	58	13	87.8	78.6	76.3	NA	63	15	82.5	79.1	75.1	NA
**Heterozygous deletion**	4	2	-	-	-		4	2	-	-	-	
**Heterozygous duplication**	1	0	-	-	-		1	0	-	-	-	
***BTG1* gene, 12q21.33 (*BTG1*-2) (N=64) (Combined categories)**							**n=69**					
**Normal**	57	13	87.6	78.2	75.8	0.695	62	15	82.3	78.8	74.6	0.824
**Abnormal**	7	2	71.4	71.4	71.4		7	2	71.4	71.4	71.4	
***BTG1* gene, 12q21.33 (*BTG1*-2) (N=64)**							**n=68**					
**Equal**	57	13	87.6	78.2	75.8	NA	62	15	82.3	78.8	74.6	NA
**Heterozygous deletion**	4	2	-	-	-		4	2	-	-	-	
**Heterozygous duplication**	3	0	-	-	-		3	0	-	-	-	
***BTG1* gene, 12q21.33 (*BTG1*-1) (N=63) (Combined categories)**							n=68					
**Normal**	57	15	84.1	76.7	72.4	0.186	62	14	79.0	75.6	69.7	0.155
**Abnormal**	6	0	100.0	100.0	100.0		6	0	100.0	100	100	
***BTG1* gene, 12q21.33 (*BTG1*-1) (N=63)**							**n=68**					
**Equal**	57	15	84.1	76.7	72.4	NA	62	18	79.0	75.6	69.7	NA
**Heterozygous deletion**	1	0	-	-	-		1	0	-	-	-	
**Heterozygous duplication**	5	0	-	-	-		5	0	-	-	-	
							1	0	-	-	-	

NA: Not applicable

SI (13): Association of *RB1* gene CNAs with EFS and OS

Factors	Event free Survival						Overall- Survival					
	No. (70)	No. of events	Cumulative Survival at			P value	No. (70)	No. of events	Cumulative Survival			P value
			1 year (%)	2 year (%)	3year (%)				1 year (%)	2 year (%)	3 year (%)	
***RB1 gene, 13q14.2 (*RB 1-6) (n=62)**							**n=68**					
**Equal**	61	17	83.5	74.7	70.7	NA	67	19	78.8	75.5	69.8	NA
**Heterozygous duplication**	1	1	-	-	-		1	1	-	-	-	
***RB1 gene, 13q14.2* (RB 1-14) (n=62)**							**n=67**					
**Equal**	60	14	86.6	79.5	75.4	NA	65	16	81.5	78.2	74.2	NA
**Heterozygous duplication**	2	1	-	-	-		2	1	-	-	-	
***RB1 gene, 13q14.2 (*RB 1-19) (n=63)**							**n=68**					
**Equal**	62	14	87.0	80.1	76.2	NA	67	16	82.1	78.9	75.0	NA
**Heterozygous duplication**	1	1	-	-	-		1	1	-	-	-	
***RB1 gene, 13q14.2 (*RB 1-24) (n=64)**							**n=69**					
**Equal**	63	16	87.2	80.5	76.6	NA	68	16	82.4	79.2	75.4	NA
**Heterozygous deletion**	1	1	-	-	-		1	1	-	-	-	
***RB1 gene, 13q14.2 (*RB 1-26) (n=64) (Combined categories)**							**n=69**					
**Normal**	60	13	88.2	81.1	77.0	NA	65	15	83.1	79.8	75.7	NA
**Abnormal**	4	2	-	-	-		4	2	-	-	-	
***RB1 gene, 13q14.2 (*RB 1-26) (n=64)**							**n=69**					
**Equal**	60	13	88.2	81.1	77.0	NA	65	15	83.1	79.8	75.7	NA
**Heterozygous deletion**	3	1	-	-	-		3	1	-	-	-	
**Heterozygous duplication**	1	1	-	-	-		1	1	-	-	-	

NA: Not Applicable

SI (14): Association of PAR1 complex genes *(Xp22.33 / Yp11.32 region)* with EFS and OS

Factors	Event free Survival						Overall- Survival					
	No. (70)	No. of events	Cumulative Survival			P value	No. (78)	No. of events	Cumulative Survival			P value
			1year (%)	2year (%)	3year (%)				1 year (%)	2year (%)	3year (%)	
***Xp22.33 / Yp11.32* region (SHOX-AREA-down) (n=58)**							**n=70**					
**Equal**	45	10	84.3	77.0	77.0	0.32	50	12	78.0	75.8	75.8	0.46
**Heterozygous duplication**	13	5	84.6	68.4	-		13	5	84.6	76.9	-	
***Xp22.33 / Yp11.32* region (CRLF2-4) (n=60)**							**n=67**					
**Equal**	44	10	86.4	79.0	76.3	0.608	49	12	77.6	77.6	75.0	0.78
**Heterozygous duplication**	16	5	87.5	74.5	-		16	5	87.5	81.3	65.7	
***Xp22.33 / Yp11.32* region [CRLF2RA-10-4] (n=61)**							**n=66**					
**Equal**	47	12	82.8	75.9	73.5	0.976	52	14	76.9	74.8	72.4	0.88
**Heterozygous duplication**	14	4	92.9	77.9	-		14	4	92.9	85.7	-	
***Xp22.33 / Yp11.32* region (IL3RA-1) (n=59) (Combined categories)**							**n=64**					
**Normal**	44	11	84.1	76.6	73.9	0.565	48	12	77.1	77.1	74.3	0.85
**Abnormal**	15	3	93.3	86.7	78.8		16	4	87.5	81.3	74.5	
***Xp22.33 / Yp11.32* region (IL3RA-1) (n=59)**							**n=64**					
**Equal**	44	11	84.1	76.6	73.9	NA	48	12	77.1	77.1	74.3	NA
**Heterozygous deletion**	1	0	-	-	-		1	0	-	-	-	
**Heterozygous duplication**	14	3	92.9	85.7	77.1		15	4	86.7	80.0	72.7	
***Xp22.33 / Yp11.32* region (P2RY8-2) (n=61) (Combined categories)**							**n=68**					
**Normal**	41	10	82.9	77.7	75.0	0.756	44	11	77.3	77.3	74.5	0.59
**Abnormal**	20	6	89.7	73.5	-		22	7	86.4	76.5	62.4	
***Xp22.33 / Yp11.32* region (P2RY8-2) (n=61)**							**n=68**					
**Equal**	41	10	82.9	77.7	75.0	0.662	44	11	77.3	77.3	74.5	0.66
**Heterozygous deletion**	1	0	-	-	-		1	0	-	-	-	
**Heterozygous duplication**	19	6	89.2	72.0	-		21	7	85.7	75.3	-	
***Xp22.33 / Yp11.32* region (ZFY-4) (n=61)**							**n=66**					
**Equal**	56	13	84.9	76.0		NA	60	15	80.0	76.4	74.3	NA
**Heterozygous duplication**	3	0	-	-			3	0	-	-	-	
**Complete deletion**	2	1	-	-			3	2	66.7	66.7	-	

SI (15): Association between Event Free Survival and demographic and clinical data.

**Data**	**Number of cases**	**Number of events**	**Cumulative survival at 1 year (%)**	**Cumulative survival at 2 year (%)**	**Cumulative survival at 3 year (%)**	**Median survival time (months)**	***P* value**
**Whole group**	70	19	84.2	76.6	71.5	NR	
**Age (n=67)**							
1-9 years	50	11	88.0	83.7	76.2	NR	0.643
≥ 10years	17	5	88.2	70.6	70.6	NR	
**Sex**							
Male	42	10	90.3	82.5	73.7	NR	0.367
Female	28	9	75.0	67.7	67.7	NR	
**Hepatomegaly**							
Positive	21	6	85.7	75.4	73.1	NR	0.731
Negative	49	13	81.0	81.0	67.9	NR	
**Splenomegaly**							
Positive	52	15	84.6	74.6	70.2	NR	0.742
Negative	18	4	83.3	83.3	76.4	NR	
**LN**							
Positive	47	11	89.4	80.1	74.7	NR	0.314
Negative	23	8	73.9	69.9	64.9	NR	
**Initial CNS***							
Positive	5	2	60.0	60.0	-	NR	0.447
Negative	65	17	86.1	77.9	72.4	NR	
**Fever**							
Positive	48	11	87.5	80.7	75.6	NR	0.276
Negative	22	8	77.3	68.2	62.9	NR	
**Anemia**							
Positive	61	15	85.2	80.0	74.1	NR	0.236
Negative	9	4	77.8	55.6	55.6	NR	
**Bleeding**							
Positive	25	7	79.6	75.2	70.7	NR	0.798
Negative	45	12	86.7	77.5	72.0	NR	

NR: Not Reached

SI (16): Association between EFS and laboratory results

Data	Number of cases	Number of events	Survival % at 1 year	Survival % at 2 year	Survival % at 3 year	Median survival time (months)	*P* value
**Total leucocytic count (/µl) ×10^9/L**							
**≤ 30**	33	7	84.4	77.2	77.2	NR	0.597
**>30**	37	11	83.8	78.4	68.7	NR	
**Hemoglobin (gm/dl)**							
**≤ 11**	65	15	86.2	81.2	75.7	NR	0.003
**>11**	5	4	60.0	-	-	13.1	
**Platelet (×10^9/L)**							
≤ 100	57	15	84.1	76.5	72.5	NR	0.852
>100	13	4	84.6	76.9	67.3	NR	
**IPT diagnosis**							
PreB	37	9	83.8	78.4	75.6	NR	NA
ProB	3	2	-	-	-	NR	
Common ALL	30	8	86.7	79.1	70.6	NR	
**CD34**							
Positive	37	6	91.8	86.2	82.7	NR	0.021
Negative	33	13	75.8	5.9	58.5	NR	
**Aberrant myeloid marker**							
Positive	6	2	83.3	66.7	-	NR	0.786
Negative	64	17	84.3	77.6	72.0	NR	
**DNR Index**							
≤ 1	53	16	81.0	73.1	68.4	NR	0.289
> 1	17	3	94.1	80.7	80.7	NR	
**IPT diagnosis**							
PreB	37	9	83.8	78.4	75.6	NR	NA
ProB	3	2	-	-	-	NR	
Common ALL	30	8	86.7	79.1	70.6	NR	

NR: Not reached NA: Not applicable

B- Supplementary figures

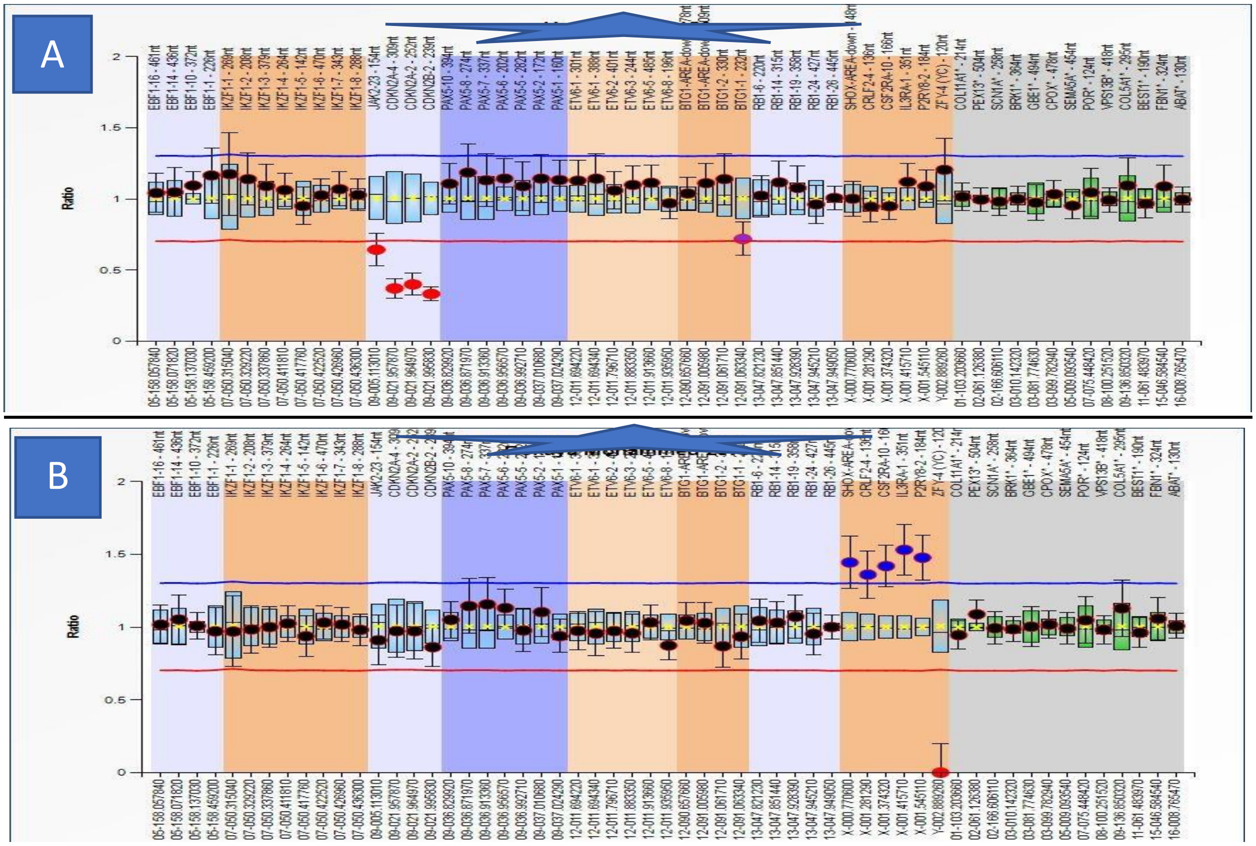

Figure 1 Two MLPA ratio charts for two of our pediatric patients showing significant CNRs. A: a case with deletion in all exons of CDKN2A/2B gene, the rest of gene exons showed normal copy number. B: a case with duplication in all exons of PAR1 complex genes (*Xp22.33 / Yp11.32 region)* and normal copy number of all other gene exons.

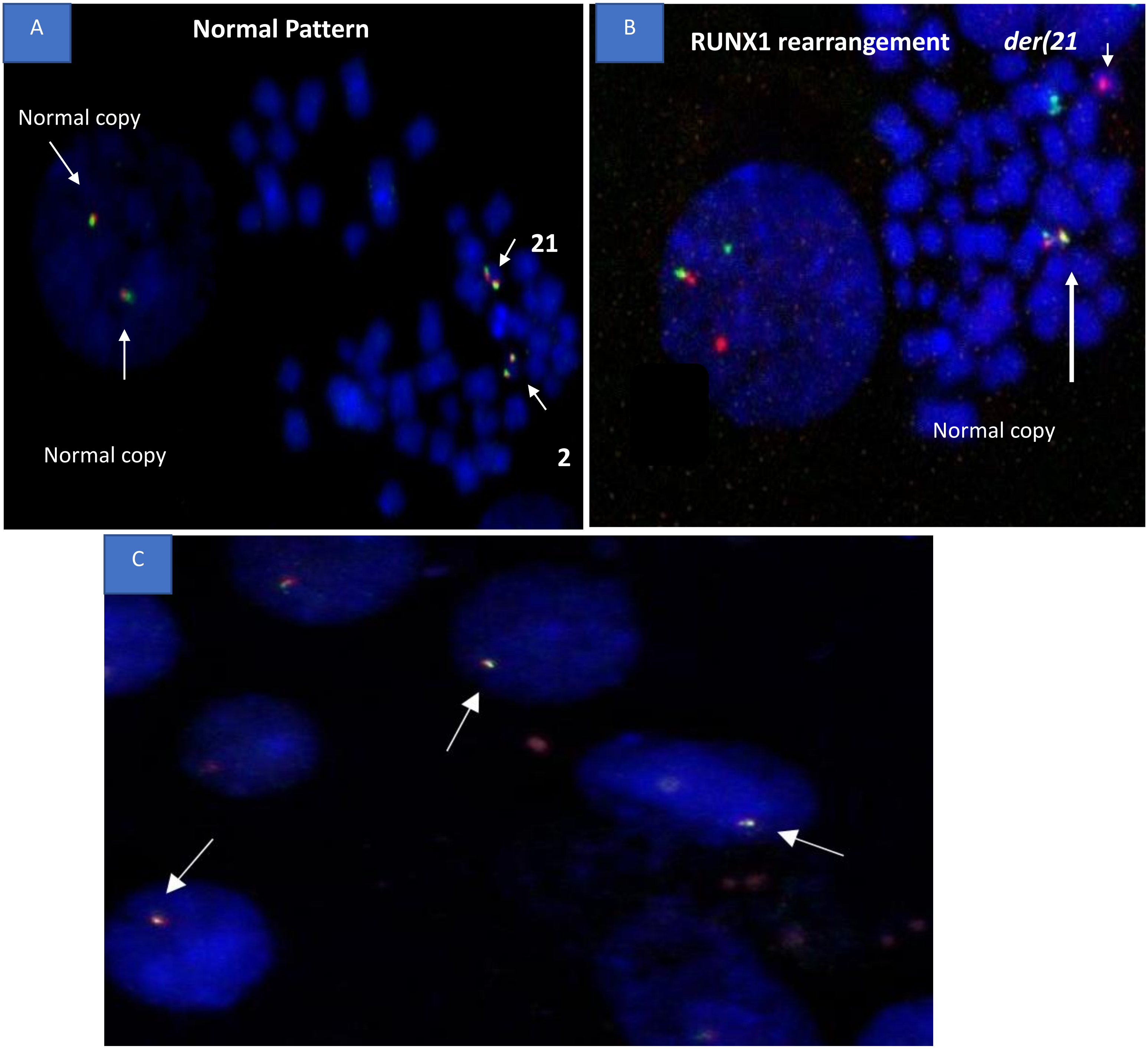

Figure 2 RUNX1 break apart FISH probe A: negative case with wild type RUNX1 gene (green and orange signals are not separated).B: positive case for RUNX1 translocation (separated orange and green signals) (C) Interphase FISH showing a single fused (orange) signal of *RUNX1* gene corresponding to *RUNX1* gene deletion. *Courtesy of Cytogenetics Unit- National Cancer Institute-Cairo University*

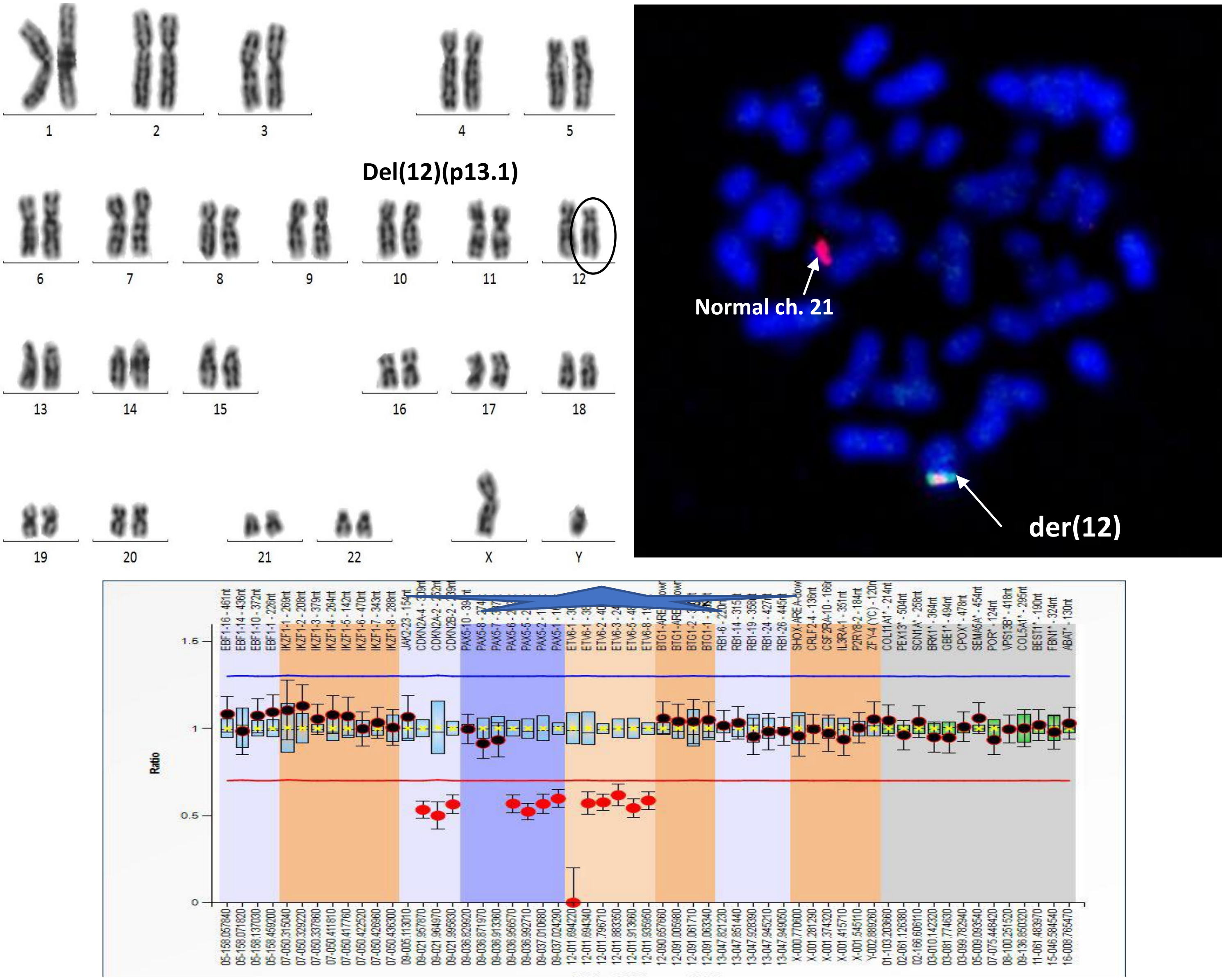

Figure 3 Karyotype, FISH and M LPA ratio chart of a BCP-ALL pediatric patient with t(12;21)(p13;q22.1); *ETV6::RUNX1* Fusion. (A) G-Banded karyotyping shows 46,XY,del(12)(p13.1),t(12;21)(p13;q22);(B) Metaphase FISH using ETV6/RUNX1 dual color translocation probe shows 1 yellow signal and 1 red signal corresponding to *ETV6::RUNX1* fusion gene and 1 copy of *RUNX1* gene respectively, confirming the loss of one copy of *ETV6* gene (green signal (C) MLPA testing revealed heterozygous deletion of *CDKN2A/2B*, heterozygous deletion of *PAX5* , and heterozygous deletion of *ETV6* in line with karyotyping and FISH results. *Courtesy of Cytogenetics Unit- National Cancer Institute-Cairo University*
